# Cdk5-mediated phosphorylation of RapGEF2 controls neuronal migration in the developing cerebral cortex

**DOI:** 10.1038/ncomms5826

**Published:** 2014-09-05

**Authors:** Tao Ye, Jacque P. K. Ip, Amy K. Y. Fu, Nancy Y. Ip

**Affiliations:** 1 Division of Life Science, The Hong Kong University of Science and Technology, Clear Water Bay, Hong Kong, China; 2 Molecular Neuroscience Center, The Hong Kong University of Science and Technology, Clear Water Bay, Hong Kong, China; 3 State Key Laboratory of Molecular Neuroscience, The Hong Kong University of Science and Technology, Clear Water Bay, Hong Kong, China

## Abstract

During cerebral cortex development, pyramidal neurons migrate through the intermediate zone and integrate into the cortical plate. These neurons undergo the multipolar–bipolar transition to initiate radial migration. While perturbation of this polarity acquisition leads to cortical malformations, how this process is initiated and regulated is largely unknown. Here we report that the specific upregulation of the Rap1 guanine nucleotide exchange factor, RapGEF2, in migrating neurons corresponds to the timing of this polarity transition. *In utero* electroporation and live-imaging studies reveal that RapGEF2 acts on the multipolar–bipolar transition during neuronal migration via a Rap1/N-cadherin pathway. Importantly, activation of RapGEF2 is controlled via phosphorylation by a serine/threonine kinase Cdk5, whose activity is largely restricted to the radial migration zone. Thus, the specific expression and Cdk5-dependent phosphorylation of RapGEF2 during multipolar–bipolar transition within the intermediate zone are essential for proper neuronal migration and wiring of the cerebral cortex.

## Abstract (web summary)

During cerebral cortex development, pyramidal neurons migrate and integrate into the cortical plate after undergoing a multipolar–bipolar transition to initiate radial migration. Here the authors show that the downstream target of Cdk5, RapGEF2, is critical for this polarity transition and the migration and integration of neurons into the cortex.

## Graphical abstract

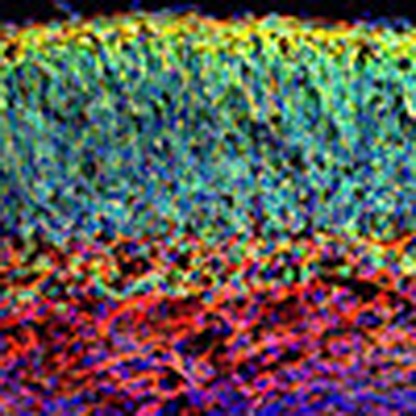

The migration of neurons from their birthplace to their final destination is fundamental to the architectural formation and functional wiring of the nervous system. In the mammalian neocortex, most pyramidal neurons are appropriately positioned in distinct cortical layers via three coordinated migration modes: multipolar migration, glia-guided locomotion and somal translocation^1^,^2^,^3^. In particular, cortical neurons first undergo multipolar migration in the lower intermediate zone before they take on a bipolar morphology in the upper intermediate zone to initiate glia-guided locomotion and radially migrate through the cortical plate. Once the migrating neurons reach the marginal zone, they position their cell bodies into their final location by somal translocation^1^. The multipolar–bipolar transition notably serves as a turning point to initiate locomotion. Perturbation of this transition disables glia-guided locomotion, which leads to cortical malformations such as periventricular heterotopia, subcortical band heterotopia and lissencephaly^4^. In turn, these conditions are associated with neuropsychiatric disorders such as epilepsy and schizophrenia^4^,^5^,^6^.

Abnormal neural architecture characterized by inverted cortical lamination^7^,^8^ has been reported in mice deficient in cyclin-dependent kinase 5 (Cdk5), a proline-directed serine/threonine kinase activated upon the association with one of its regulatory subunit proteins, p35 or p39 (ref. 9). Although several Cdk5 substrates regulate glia-guided locomotion via leading process dynamics (for example, Pak1 and p27Kip1)^10^,^11^ and nucleokinesis (for example, Nudel, DCX and FAK)^12^,^13^,^14^, recent evidence suggests that Cdk5 may begin to exert its function in multipolar–bipolar transition to induce locomotion^15^. Nonetheless, the *in vivo* downstream target(s) that mediates Cdk5 function in multipolar–bipolar transition remains unclear.

Rap1 signalling is involved in neuronal migration, and is implicated to be regulated by Cdk5 (ref. 16). Activation of Rap1 depends on its specific guanine nucleotide exchange factors (GEFs) and RapGEF1 (also known as C3G) is reported to control somal translocation triggered by Reelin^17^,^18^,^19^. Another Rap1 GEF—RapGEF2 (also called PDZ-GEF1/RA-GEF1)^20^,^21^,^22^—contains several domains including cyclic nucleotide-binding, Ras exchange, PDZ, Ras association (RA) and Rap GEF domains as well as two E3 ligase-binding PY motifs and one PDZ-binding motif^23^. The GEF domain of RapGEF2 efficiently stimulates GTP exchange on both Rap1 and its close family member, Rap2 (ref. 20). Other domains appear to regulate its activity, stability and localization^22^,^23^,^24^. Importantly, RapGEF2-deficient mice exhibit heterotopic bands in the subcortical area^25^, implicating its role in early brain development. Furthermore, RapGEF2-mediated Rap1 activation is implicated in other processes of neuronal morphogenesis^26^,^27^. Despite the potential significance of RapGEF2 in brain development, surprisingly little is known about its regulation in the developing brain. It is also unclear whether the two RapGEFs, C3G and RapGEF2 have redundant or specific function in different cellular events during brain development, and how their activity is precisely controlled.

The present study demonstrates the cell-autonomous function of RapGEF2 in neuronal migration during cortical development. *In utero* electroporation and live-imaging studies reveal that the specific activation of Rap1/N-cadherin pathway by RapGEF2 in the intermediate zone is required for multipolar–bipolar transition during neuronal migration. In contrast to other neuronal migration regulators expressed ubiquitously in the intermediate zone, RapGEF2 is preferentially found in the radial migration zone and not the multipolar migration zone. Importantly, the activity of RapGEF2 increases upon phosphorylation by Cdk5, whose kinase activity is also largely restricted to the radial migration zone. Thus, the precise control of RapGEF2 activity through its specific expression and Cdk5-dependent phosphorylation is critical for proper neuronal and cortical circuit assembly.

## Results

### RapGEF2 is developmentally regulated in the neocortex

**Figure 1:**
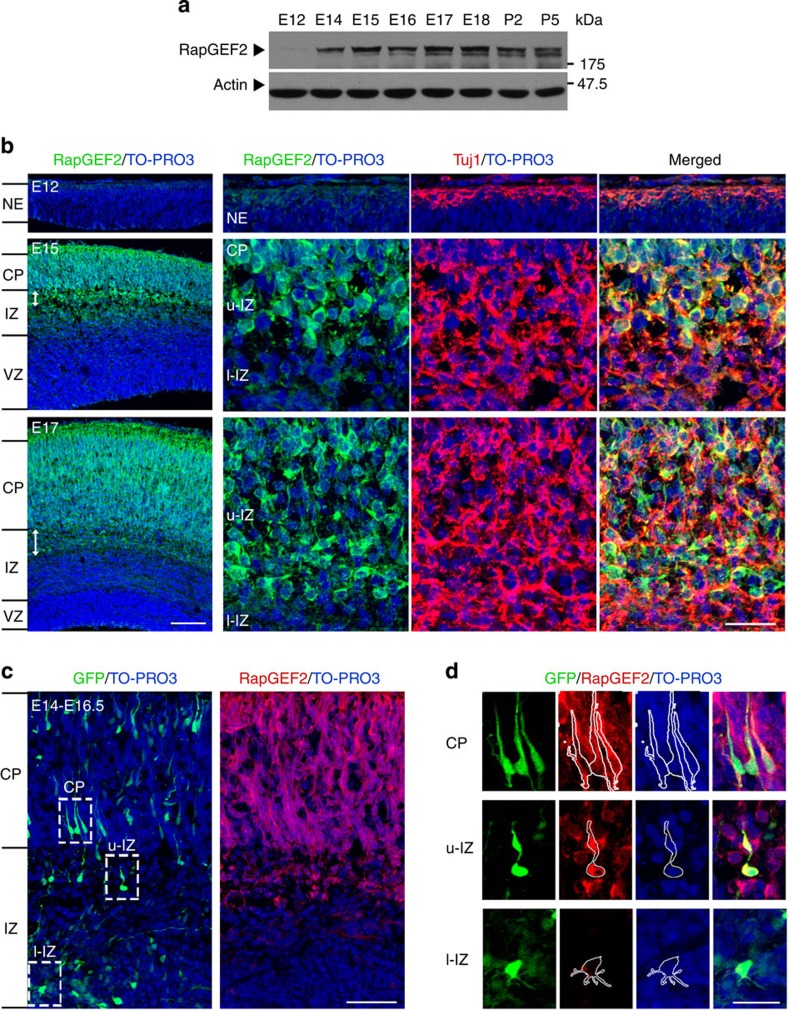
Developmental expression of RapGEF2 in the neocortex. (**a**) Temporal expression of RapGEF2 protein in the mouse neocortex during embryonic and early postnatal development. Actin served as the loading control. See full-length blots in Supplementary Fig. 10. (**b**) Dynamic spatial expression pattern of RapGEF2 in the cerebral cortex during development. Coronal brain sections at embryonic days (E) 12, 15 and 17 were collected and stained for RapGEF2, Tuj1 (a neuronal marker) and TO-PRO3 (a nuclear marker). Double-headed arrows, upper intermediate zone (u-IZ). NE, neuroepithelium; VZ, ventricular zone; l-IZ, lower intermediate zone; CP, cortical plate. Scale bars, 100 and 20 μm (left and right panels, respectively). (**c**) E14 mouse brains were electroporated with GFP plasmid. Representative E16.5 (60 h post electroporation) cortical sections were stained by GFP, RapGEF2 and TO-PRO3. Scale bar, 100 μm. The experiment was repeated for at least three times. (**d**) Magnified images of selected regions in **c**. Scale bar, 20 μm.

To study the function of RapGEF2 in neuronal migration, we first examined its spatial and temporal expression profiles in the developing mouse cortex (Fig. 1a–d). In western blot analyses of mouse cortex extracts at different developmental stages (embryonic day (E) 12 to postnatal day (P) 5), RapGEF2 was barely detectable in E12 mouse cortices. Its expression was upregulated during the critical stages of neuronal migration in the developing neocortex and remained high thereafter (Fig. 1a). To detect the spatial expression pattern of RapGEF2 in the cortical wall of developing mouse brains, we examined coronal cortical sections from mouse embryos collected at E12, E15 and E17. RapGEF2 was barely detected at E12 neocortex, which mainly comprises neural progenitor cells. At E15 and E17, when robust neuronal migration occurs, RapGEF2 expression was prominently upregulated in nascent migrating neurons located in the upper intermediate zone. In particular, RapGEF2 was mainly localized in the cytoplasm but absent from the nucleus^25^ (Fig. 1b). *In utero* electroporation was used to further visualize migrating neurons in the developing cortex, revealing that RapGEF2 was upregulated precisely when migrating neurons obtained a bipolar morphology in the upper intermediate zone (Fig. 1c,d). These findings prompted us to investigate whether the dynamic regulation of RapGEF2 during multipolar–bipolar transition is essential for migrating neurons to pass through the intermediate zone and integrate into their destined cortical layers.

### RapGEF2 regulates neuronal migration to the cortical plate

**Figure 2:**
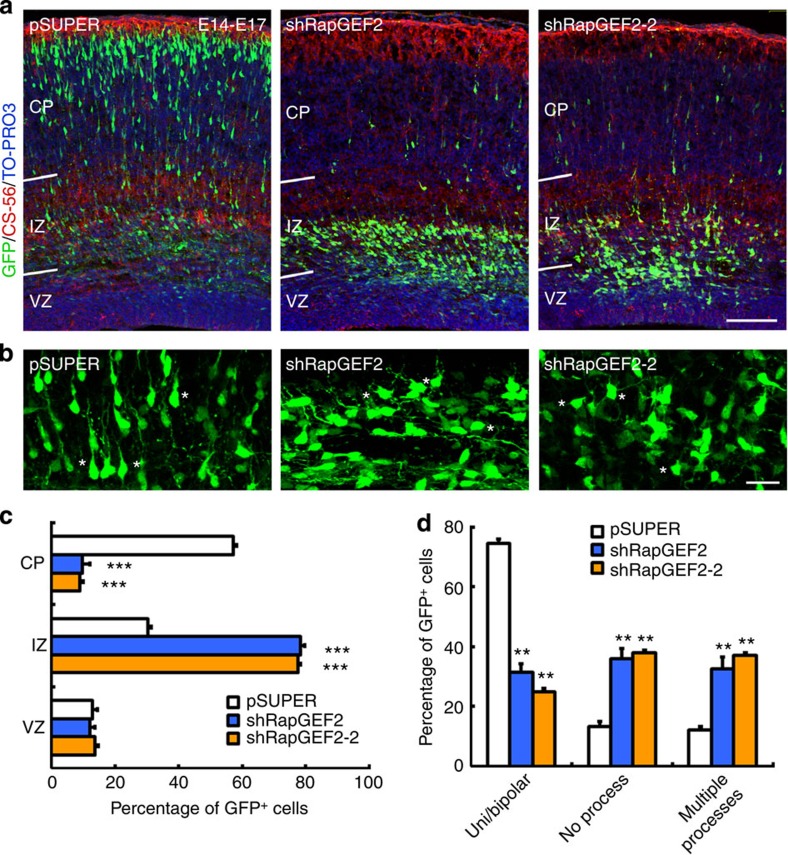
RapGEF2 regulates neuronal migration to the cortical plate. (**a**) E14 mouse brains were co-electroporated with vector control (pSUPER) or two different RapGEF2 shRNAs (shRapGEF2 or shRapGEF2-2) together with GFP plasmid. Representative E17 cortical sections were stained by GFP, CS-56 (a subplate marker) and TO-PRO3. Scale bar, 100 μm. The experiment was repeated for at least three times. (**b**) Magnified images of individual electroporated neurons with comparable GFP^+^ cell density in the intermediate zone after RapGEF2 knockdown. Asterisks indicate representative neurons in each group. Scale bar, 20 μm. (**c**) Quantification of the percentages of GFP^+^ neurons in different cortical layers. Error bars indicate the s.e.m. of five different brains containing >1,000 neurons. (**d**) Quantification of the percentages of neurons with uni- or bipolar morphology, no process and multiple (≥3) processes. Error bars indicate the s.e.m. of three different brains containing >120 neurons. *P<0.05, **P<0.01, ***P<0.001 versus pSUPER; Student’s *t*-test.

The restrictive expression of RapGEF2 in the upper intermediate zone during cortical development prompted us to examine its functional importance in neuronal migration by acutely abrogating its expression in mouse embryos through *in utero* electroporation. Two short hairpin RNAs (shRNAs; shRapGEF2 and shRapGEF2-2) that recognize distinct regions of RapGEF2 transcript were used to examine the effect of RapGEF2 suppression during neuronal migration. RapGEF2 shRNA knocked down endogenous RapGEF2 expression in primary cultured cortical neurons and migrating neurons *in vivo* (Supplementary Fig. 1a–c). RapGEF2 knockdown disrupted the migration of projection neurons during neocortical development. Most green fluorescent protein (GFP)-labelled neurons in the pSUPER vector or scrambled shRNA groups migrated through the intermediate zone and reached the upper region of the cortical plate at E17 (Supplementary Movie 1). However, most GFP^+^ neurons in RapGEF2-knockdown brains expressing either shRapGEF2 or shRapGEF2-2 failed to leave the lower intermediate zone and accumulated beneath the CS-56-labelled upper intermediate zone (Fig. 2a,c; Supplementary Fig. 1d,f; Supplementary Movie 2). Detailed examination of cell morphology showed that most control neurons had a characteristic bipolar morphology with one major leading process oriented towards the cortical plate. Conversely, most RapGEF2-knockdown neurons arrested in the intermediate zone failed to undergo multipolar–bipolar transition and exhibited abnormal morphologies with either multiple short processes or no neuronal process (Fig. 2b,d; Supplementary Fig. 1e,g).

The results confirm that RapGEF2 suppression does not affect the morphological integrity of nestin-labelled radial glial fibres, the number of Pax6^+^ apical or Tbr2^+^ intermediate progenitor cells, nor caspase-3-induced neuronal cell death (Supplementary Fig. 2a–f). These results strongly suggest that RapGEF2 is required for multipolar–bipolar conversion and neuronal migration. Furthermore, the observed phenotype on RapGEF2 suppression is not the secondary effect of defects in other developmental processes.

Because neurons generated from E14 integrate into the upper layers (that is, layers II–IV) of the cerebral cortex, we speculated that RapGEF2 controls the migration of newborn neurons that assemble to form different cortical layers. To this end, RapGEF2 shRNA was introduced into the lateral cortex by *in utero* electroporation at E12, when Ctip2^+^ layer V neurons are generated. These RapGEF2-suppressed neurons were similarly positioned within the intermediate zone and failed to enter the cortical plate 3 days after electroporation (Supplementary Fig. 3a,b). This result suggests that RapGEF2 function is essential for the multipolar–bipolar transition and migration of neurons from different cortical layers.

There is a neurogenic gradient between the lateral cortex and the dorsomedial cortex (that is, the former is older than the latter). Accordingly, electroporation of the lateral cortex at E12 labels those neurons that pass through the multipolar migration phase and mainly use locomotion^2^,^28^, whereas electroporation of the dorsomedial cortex labels the neurons that only use somal translocation^29^. In the present study, electroporation of RapGEF2 shRNA into the dorsomedial cortex did not affect neuronal migration (Supplementary Fig. 3c,d), suggesting that RapGEF2 is not required in Tbr1^+^ layer VI neurons for somal translocation.

### RapGEF2 regulates multipolar–bipolar transition

**Figure 3:**
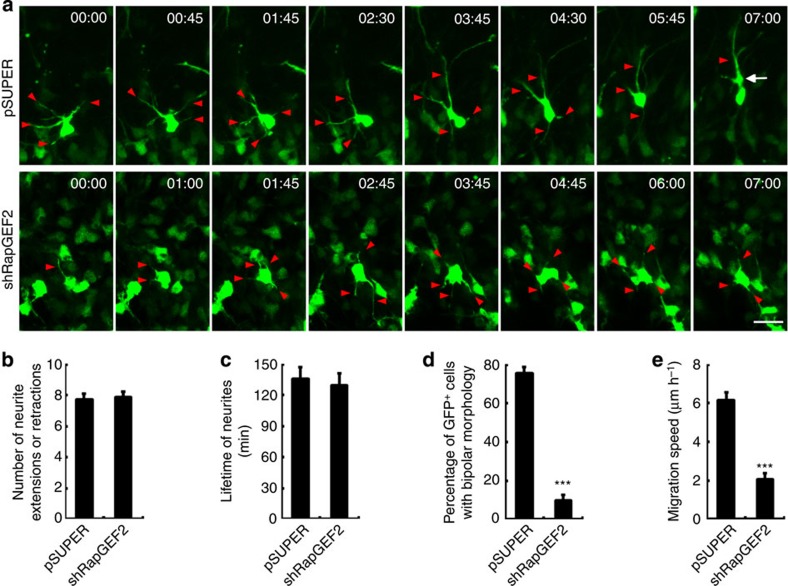
RapGEF2 is essential for multipolar–bipolar transition. (**a**) E14 mouse brains were electroporated *in utero* using control (pSUPER) or RapGEF2 shRNA (shRapGEF2) together with GFP plasmid. Living cortical slices were prepared at E16 and the migratory behaviours of GFP^+^ neurons were imaged for 7 h. Red arrowheads, neurites extending from cell bodies. White arrowhead, swelling of leading process of a control neuron. Scale bar, 20 μm. (**b**) Quantification of the number of neurite extension and retraction events of GFP^+^ cells. Error bars indicate the s.e.m. of six different brains containing 20 GFP^+^ cells in each group. (**c**) Quantification of the neurite lifetime of GFP^+^ cells. Error bars indicate the s.e.m. of six different brains containing 20 GFP^+^ cells with >60 neurites in each group. (**d**) Percentage of GFP^+^ cells transiting to bipolar morphology in the imaging period. Error bars indicate the s.e.m. of six different brains containing >60 neurons. (**e**) Quantification of the migration speed of GFP^+^ cells. Error bars indicate the s.e.m. of six different brains containing 20 GFP^+^ cells. ***P<0.001 versus pSUPER; Student’s *t*-test.

Before radial locomotion in the cortical plate, migrating neurons undergo a transient multipolar migration phase in the intermediate zone in which they exhibit random active neurite extension and retraction, and subsequently acquire a radially oriented bipolar morphology. To directly determine whether RapGEF2 is critical for multipolar–bipolar transition, we performed time-lapse analysis of living cortical slices and observed the migratory behaviour of individual pSUPER- or RapGEF2 shRNA-electroporated neurons. Control neurons exhibited active neurite extension and retraction, and eventually acquired a radially oriented leading process towards the pial surface (Fig. 3a; Supplementary Movie 3). Conversely, RapGEF2-suppressed neurons exhibit impaired bipolar transition and reduced migration speed (Fig. 3d,e; Supplementary Movie 4). Nevertheless, RapGEF2 knockdown does not regulate neurite dynamics, because neither the number of neurite extension or retraction events nor average lifetime of extended neurites was affected (Fig. 3b,c). Thus, these observations indicate that RapGEF2 regulates the formation of a radially oriented leading process, probably through a cellular mechanism besides neurite dynamics.

### RapGEF2 knockdown causes accumulation of ectopic neurons

**Figure 4:**
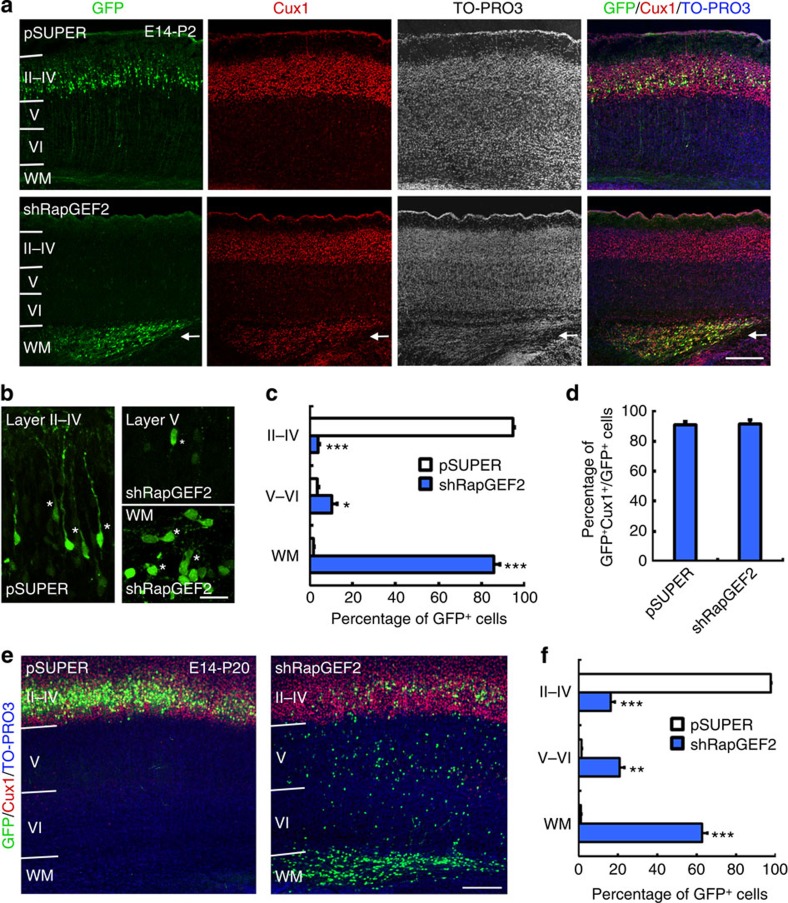
RapGEF2 knockdown causes accumulation of ectopic neurons. (**a**) E14 mouse brains were electroporated *in utero* using control (pSUPER) or RapGEF2 shRNA (shRapGEF2) together with GFP plasmid. Representative postnatal day (P) 2 cortical sections were stained for GFP, Cux1 (a layer II–IV neuron marker) and TO-PRO3. Arrows denote the heterotopic band of arrested neurons. Scale bar, 200 μm. The experiment was repeated for at least three times. (**b**) GFP^+^ neurons at higher magnification. Asterisks indicate representative neurons. Scale bar, 20 μm. (**c**) Quantification of the percentages of pSUPER- and RapGEF2 shRNA-electroporated neurons in different cortical layers. Error bars indicate the s.e.m. of five different brains containing >1,000 GFP^+^ cells. (**d**) Quantification of Cux1^+^ neurons. Error bars indicate s.e.m. More than 600 cells were analysed from three brains in each group. (**e**) P20 brain sections were stained for GFP, Cux1 and TO-PRO3. Scale bar, 200 μm. (**f**) Quantification of the percentages of GFP^+^ neurons in different cortical layers at P20. Error bars indicate the s.e.m. of five different brains containing >1,000 neurons. *P<0.05, **P<0.01, ***P<0.001 versus pSUPER; Student’s *t*-test.

To determine whether defective migration induced by RapGEF2 shRNA temporarily or permanently affects cortical lamination, we examined the cerebral cortex 6 days post electroporation at P2. Most control neurons migrated up to the cortical plate and subsequently settled in cortical layers II–IV (Fig. 4a). Meanwhile, most RapGEF2-suppressed neurons remained accumulated in the intermediate zone, leading to the malformation of an ectopic neuronal layer beneath the cortical plate positive for Cux1, a layer II–IV marker (Fig. 4c,d). Furthermore, the RapGEF2-depleted neurons at P2 exhibited morphological defects similar to those at E17, including shortened leading processes in the cortical layers and a lack of polarized morphology (that is, unipolar/bipolar) in the white matter (Fig. 4b).

To determine the long-term consequences of RapGEF2 depletion at E14, we examined the mouse cerebral cortex at P20, when neuronal migration is normally completed. In vector-electroporated brains, almost all GFP^+^ neurons were positioned in layers II–IV of the mature cortex and exhibited an extensively differentiated dendritic arbour, suggesting proper pyramidal neuron differentiation (Fig. 4e; Supplementary Fig. 4). RapGEF2 knockdown strikingly resulted in a heterotopic band of neurons in the subcortical white matter, which corresponds to the intermediate zone in the developing cortex (Fig. 4e,f). Thus, neurons with suppressed RapGEF2 expression permanently failed to migrate up and integrate into the proper cortical layers. Notably, even though a few RapGEF2-knockdown neurons migrated into the superficial layers II–IV, their dendrite arbourization was severely perturbed on RapGEF2 depletion (Supplementary Fig. 4). Further characterization of these trapped RapGEF2-knockdown neurons revealed a defective phenotype similar to that observed during earlier developmental stages (E17 and P2), that is, a severely perturbed morphology characterized by a distorted cell body shape and abnormal dendritic morphogenesis. Furthermore, some RapGEF2 shRNA-electroporated neurons in the white matter were round with several minor processes (Supplementary Fig. 4).

### Exit from multipolar phase requires RapGEF2 activity

**Figure 5:**
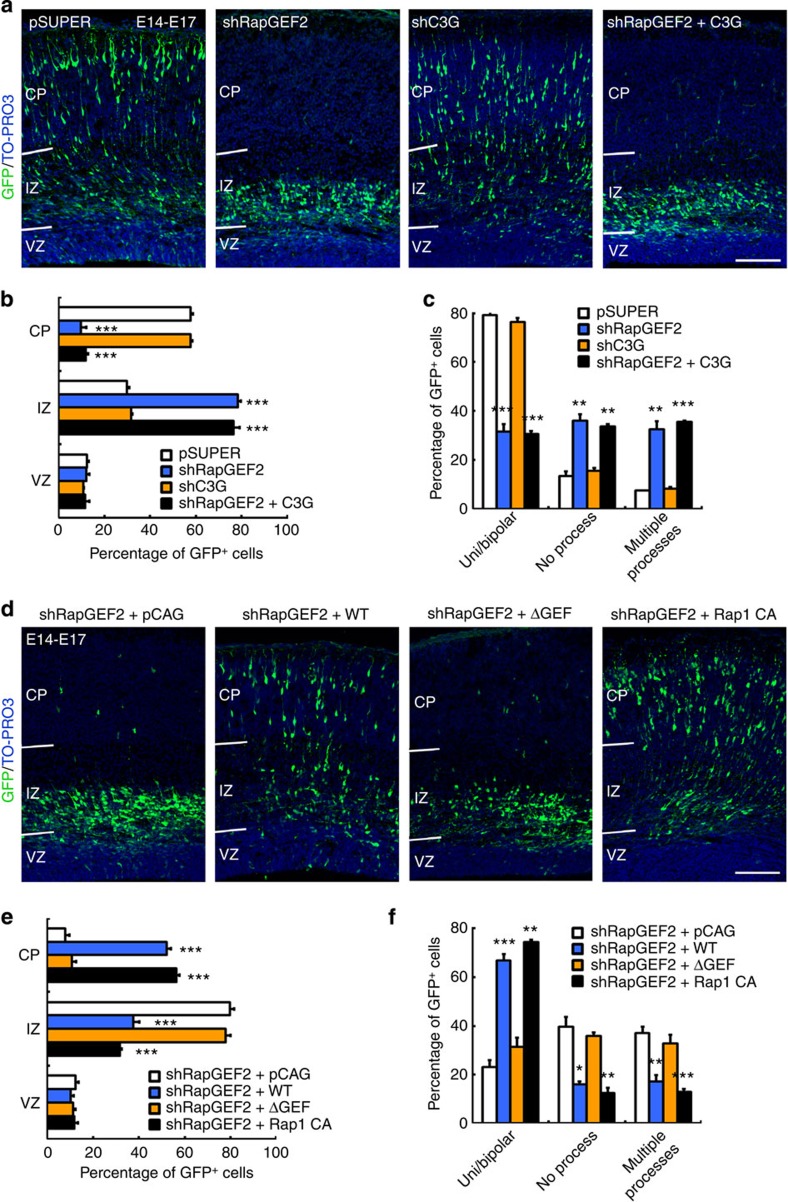
Neuronal migration requires the Rap1 activity of RapGEF2. (**a**) E14 cerebral cortices electroporated with vector control (pSUPER) or shRNAs targeting RapGEF2 or C3G (shRapGEF2 or shC3G) together with GFP plasmid, or shRapGEF2 plus C3G-expressing plasmids were examined at E17. Representative cortical sections at E17 were stained for GFP and TO-PRO3. Scale bar, 100 μm. The experiment was repeated for at least three times. (**b**) Quantification of the percentages of GFP^+^ neurons in different cortical layers. Error bars indicate the s.e.m. of four different brains containing >600 neurons. (**c**) Quantification of the percentages of neurons with uni- or bipolar morphology, no process and multiple (≥3) processes. Error bars indicate the s.e.m. of three different brains containing >120 neurons. **P<0.01, ***P<0.001 versus pSUPER; Student’s *t*-test. (**d**) Representative cerebral cortices at E17 electroporated with the indicated combinations of plasmids at E14 were stained for GFP and TO-PRO3. Scale bar, 100 μm. The experiment was repeated for at least three times. (**e**) Quantification of the percentages of GFP^+^ neurons in different cortical layers. Error bars indicate the s.e.m. of five different brains containing >1,000 neurons. (**f**) Quantification of the percentages of neurons with uni- or bipolar morphology, no process and multiple (≥3) processes. Error bars indicate the s.e.m. of three different brains containing >120 neurons. *P<0.05, **P<0.01, ***P<0.001 versus shRapGEF2+pCAG group; Student’s *t*-test.

The small GTPase Rap1 has been suggested to play important roles in multiple steps of neuronal migration, including multipolar–bipolar transition and somal translocation^17^,^18^. However, the spatiotemporal regulation of Rap1 activity during distinct migration phases remains unclear. Among the several Rap1 GEFs, C3G and RapGEF2 are thought to be required for neuronal migration^25^,^30^. To determine whether Rap1-mediated multipolar–bipolar transition is specifically controlled by RapGEF2, we suppressed the expression of Rap1 GEFs at E14 using C3G shRNA^31^ (Supplementary Fig. 5a,b) or RapGEF2 shRNA, respectively, and analysed migration defects at E17. As mentioned earlier, RapGEF2 knockdown resulted in neuronal mislocalization and arrest in the multipolar cell phase (Figs 2a,c,d and 5a–c). Meanwhile, C3G knockdown neither impaired the bipolar transition nor delayed neuronal migration into the cortical plate at E17 (Fig. 5a,c,d); instead, it significantly reduced the percentage of GFP^+^ cells migrating into the superficial region of the cerebral cortex at P0, which is indicative of a defect in the late phase of neuronal migration (Supplementary Fig. 5c,d). These results are concordant with those of a recent study suggesting that C3G pathway is required for terminal translocation^19^. Furthermore, C3G expression did not restore neuronal migration in RapGEF2-knockdown cortices (Fig. 5a–c). Together, these findings indicate that neurons specifically require RapGEF2 but not C3G to exit the multipolar cell phase and migrate into the cortical plate. Thus, although both GEFs can regulate Rap1 activity, they play specific roles in different cellular events during neuronal migration.

Then, we determined whether the GEF activity of RapGEF2 is required for its function in neuronal migration. The re-expression of wild-type RapGEF2 (WT), but not the GEF domain-deletion mutant of RapGEF2 (ΔGEF), restored the defective bipolar transition and migration of neurons into the cortical plate (Fig. 5d–f; Supplementary Fig. 6a). These results suggest that proper neuronal migration requires the GEF activity of RapGEF2. Furthermore, ectopic expression of constitutively active Rap1 (Rap1 CA)^32^ increased Rap1 activity and reversed the migration defects induced by RapGEF2 knockdown (Fig. 4f–h). Thus, the replenishment of active Rap1 protein indeed restores normal neuronal migration in RapGEF2-knockdown cortices. The expression of RapGEF2 proteins or constitutively active Rap1 without RapGEF2 shRNA led to similar results to the rescue experiments (Supplementary Fig. 6b,c). These findings collectively show that RapGEF2-dependent Rap1 activation during bipolar transition is a critical molecular event that safeguards proper neuronal migration *in vivo*.

### Cdk5 phosphorylates RapGEF2 at Ser1124 *in vivo*

**Figure 6:**
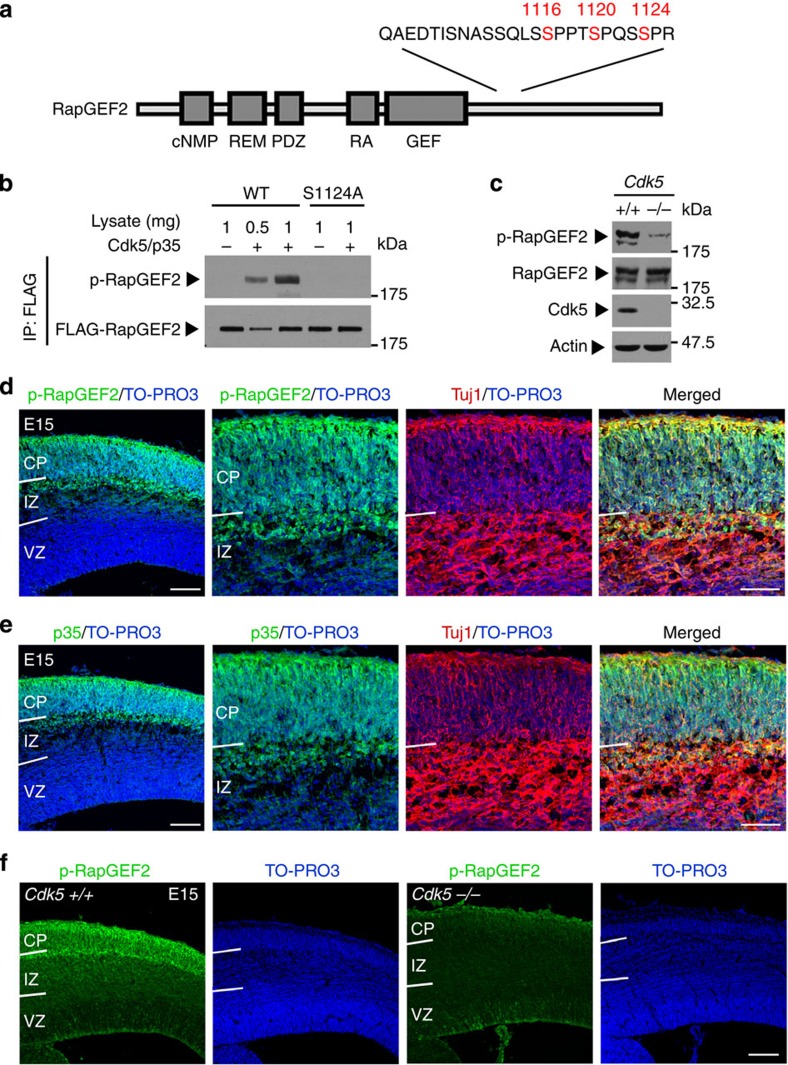
RapGEF2 is an *in vivo* substrate of Cdk5/p35. (**a**) Diagram of the domain structure of RapGEF2 protein. The phosphopeptide identified by the phosphoproteomic analysis is shown. The three proline-directed serine sites are highlighted. (**b**) Validation of p-RapGEF2 antibody in HEK293T cells. (**c**) Absence of RapGEF2 phosphorylation at Ser1124 from Cdk5-deficient cortices at E18. Actin served as the loading control. The experiment was repeated for at least three times. See full-length blots in Supplementary Fig. 10. (**d**) Spatial expression of p-RapGEF2 in the developing mouse cortex. Representative E15 cortical sections were co-stained for p-RapGEF2, Tuj1 and TO-PRO3. The experiment was repeated for at least three times. (**e**) Spatial expression of p35 in the developing mouse cortex. Representative E15 cortical sections were co-stained for p35, Tuj1 and TO-PRO3. The experiment was repeated for at least three times. (**f**) Wild-type or Cdk5-deficient cortical sections at E15 were stained for p-RapGEF2 and TO-PRO3. Scale bar, 100 μm (**d**–**f**).

Next, we investigated the underlying mechanism by which RapGEF2 is activated during neuronal migration. In an independent study, we attempted to identify novel Cdk5 substrates using mass spectrometry and revealed that a phosphopeptide with amino-acid sequence derived from RapGEF2 is decreased in the brains of Cdk5-conditional knockout mice^33^ (Ip *et al*., unpublished observations). Cdk5 preferentially phosphorylates substrates at the Ser/Thr sites within a consensus motif, (S/T)PX(K/H/R)^34^; this RapGEF2 phosphopeptide contains one strong consensus Cdk5 phosphorylation motif, SPRK, residing at Ser1124 (Fig. 6a). Together with the functional similarity between Cdk5 and RapGEF2 in multipolar–bipolar transition, these findings warranted investigating whether Cdk5 phosphorylates RapGEF2 at Ser1124 to regulate RapGEF2 function in neuronal migration. FLAG-tagged RapGEF2-ct, a carboxy terminus Ser-rich fragment of RapGEF2 that contains Ser1124 and other proline-directed serine sites, was phosphorylated by Cdk5/p35 complex in an *in vitro* phosphorylation assay (Supplementary Fig. 7a). In addition, the mutation of RapGEF2-ct Ser1124 to alanine (S1124A, S1116/1124A, S1120/1124A or S1116/1120/1124A) substantially reduced RapGEF2-ct phosphorylation, whereas mutation of other serine residues to alanine (S1116A, S1120A or S1022/1080/1226A) barely reduced it (Supplementary Fig. 7a). These results suggest that RapGEF2 is predominantly phosphorylated by Cdk5 at Ser1124. To confirm that RapGEF2 is a substrate for Cdk5, we generated a phospho-specific RapGEF2 antibody, p-RapGEF2, against the epitope containing the phospho-Ser1124 residue of RapGEF2. The Cdk5/p35 complex phosphorylated full-length RapGEF2 at Ser1124 in an *in vitro* phosphorylation assay. Furthermore, no Ser1124-phorphorylated RapGEF2 was detected in the absence of Cdk5/p35 or when phosphodeficient RapGEF2 (S1124A) was expressed, whereas RapGEF2 (WT) was phosphorylated by recombinant Cdk5/p35 complex in a dose-dependent manner (Fig. 6b). This demonstrates that Cdk5 phosphorylates RapGEF2 at Ser1124 *in vitro*. To determine if RapGEF2 is a Cdk5 substrate *in vivo*, RapGEF2 phosphorylation at Ser1124 was examined in the brain lysates derived from Cdk5^−/−^ mice and wild-type littermates. Similar to that of RapGEF2, phospho-RapGEF2 antibody detected double bands in wild-type mouse brains. Conversely, phospho-RapGEF2 was dramatically decreased in Cdk5-deficient brains, although the total protein levels of RapGEF2 remained relatively unchanged (Fig. 6c). Thus, RapGEF2 is phosphorylated by Cdk5 in the developing mouse cerebral cortex.

To determine the specificity and spatial expression pattern of phospho-RapGEF2 in the mouse cortex during neuronal migration, we stained coronal cortical sections from mouse embryos collected at E15 with phospho-RapGEF2 antibody. The staining pattern of phospho-RapGEF2 was similar to that of total RapGEF2 protein, showing restrictive signals in the upper intermediate zone and cortical plate as well as co-localization with neuronal marker Tuj1 (Fig. 6d). The phospho-staining pattern was abolished when the phospho-RapGEF2 antibody was pre-absorbed with the phosphopeptide antigen; this indicates that the antibody specifically recognizes phospho-RapGEF2 (Supplementary Fig. 7b). In addition, p35, the major Cdk5 activator during brain development, exhibited a similar restrictive distribution pattern as that of phospho-RapGEF2 in the developing cerebral cortex (Fig. 6e; Supplementary Fig. 7c). Importantly, immunohistochemical analysis of E15 cortical sections from wild-type and Cdk5-deficient mice showed that RapGEF2 phosphorylation at Ser1124 was barely detected in the intermediate zone or cortical plate of Cdk5-deficient cortices (Fig. 6f); these results suggest that this phosphorylation event plays an essential role in Cdk5-dependent multipolar–bipolar transition.

### RapGEF2 phosphorylation controls neuronal migration via Rap1

**Figure 7:**
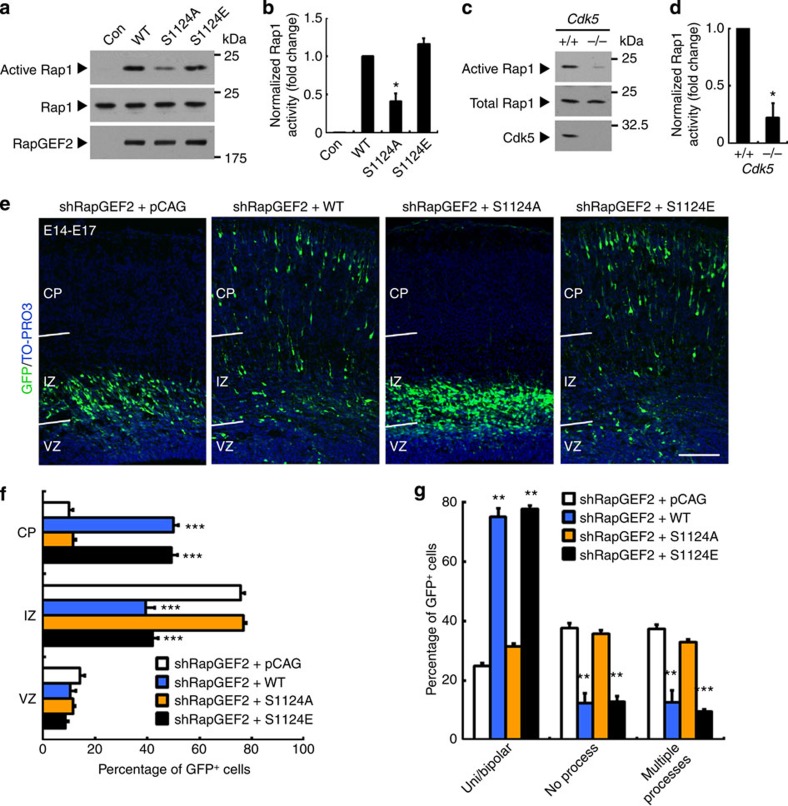
RapGEF2 phosphorylation controls neuronal migration via Rap1. (**a**) Pull-down assay of active Rap1 in the lysates of HEK293T cells expressing vector control (Con), wild type (WT), phosphodeficient (S1124A) or phosphomimetic (S1124E) mutants of RapGEF2 using GST-RalGDS-RBD. (**b**) Quantification of fold change of active Rap1 levels. Error bars indicate the s.e.m. of four independent experiments. *P<0.05 versus WT-expressing group; Student’s *t*-test. (**c**) Pull-down assay of active Rap1 in the cortical lysates from E18 Cdk5^−/−^ mice and their corresponding wild-type littermates using GST-RalGDS-RBD. See full-length blots in Supplementary Fig. 10. (**d**) Quantification of fold change of active Rap1 levels. Error bars indicate the s.e.m. of three independent experiments. (**e**) Co-electroporation of E14 mouse brains was performed using RapGEF2 shRNA (shRapGEF2) together with control (pCAG) or the indicated RapGEF2-expressing plasmids (WT, S1124A or S1124E). E17 coronal cortical sections were stained for GFP and TO-PRO3. Scale bar, 100 μm. (**f**) Quantification of the percentages of GFP^+^ cells expressing control or different RapGEF2 constructs in different cortical layers at E17. Error bars indicate the s.e.m. of four different brains containing >600 neurons. (**g**) Quantification of the percentages of GFP^+^ neurons with uni- or bipolar morphology, no process and multiple (≥3) processes. Error bars indicate the s.e.m. of three different brains containing >120 neurons. *P<0.05, **P<0.01, ***P<0.001 versus shRapGEF2+pCAG group; Student’s *t*-test.

Next, we examined how Cdk5-mediated phosphorylation of RapGEF2 regulates its GEF activity towards Rap1. RapGEF2 (WT) or mutants in which the phosphorylation site was changed to alanine or glutamate to mimic nonphosphorylated or phosphorylated states, respectively, were expressed in HEK293T cells. A glutatione S-transferase (GST) fusion protein containing the Rap-binding domain of RalGDS (GST-RalGDS-RBD) was used to measure Rap1 activity. Mutation of Ser1124 to alanine resulted in a significantly lower level of active Rap1 (Fig. 7a,b), indicating that Cdk5-mediated phosphorylation is essential for the GEF activity of RapGEF2 towards Rap1. To determine whether Cdk5 regulates Rap1 activation *in vivo* during the developmental stage of neuronal migration, we measured Rap1 activity in E18 Cdk5-knockout brains. Concordant with our hypothesis, active Rap1 levels were significantly lower in the cortex of Cdk5-deficient mice than their wild-type littermates (Fig. 7c,d). These results collectively suggest that Cdk5 is involved in Rap1 activation during cortical development.

As mentioned above, the GEF activity of RapGEF2 is specifically required for multipolar–bipolar transition and neuronal entry into the cortical plate during neuronal migration. To study the regulatory role of the Cdk5-mediated RapGEF2 phosphorylation in neuronal migration, various RapGEF2 mutants were re-expressed in RapGEF2-depleted cortices and their effects on the restoration of proper neuronal migration were examined (Fig. 6e; Supplementary Fig. 6a). Introducing a phosphomimetic mutant of RapGEF2, S1124E, restored multipolar–bipolar transition and migration to the similar extent as that achieved by RapGEF2 (WT) re-expression. However, the re-expression of the phosphodeficient mutant of RapGEF2, S1124A, which exhibited reduced Rap1 activity *in vitro*, failed to rescue the defective multipolar–bipolar transition or migration of cortical neurons caused by RapGEF2 depletion (Fig. 7e–g). The expression of these RapGEF2 mutants without RapGEF2 shRNA produced results similar to those of rescue experiments (Supplementary Fig. 6b,c). Finally, shRNA-mediated suppression of Cdk5 resulted in migration defects that phenocopied the RapGEF2-knockdown effects and were ameliorated by the expression of S1124E but not S1124A of RapGEF2 (Supplementary Fig. 8).

### RapGEF2 phosphorylation regulates N-cadherin function

**Figure 8:**
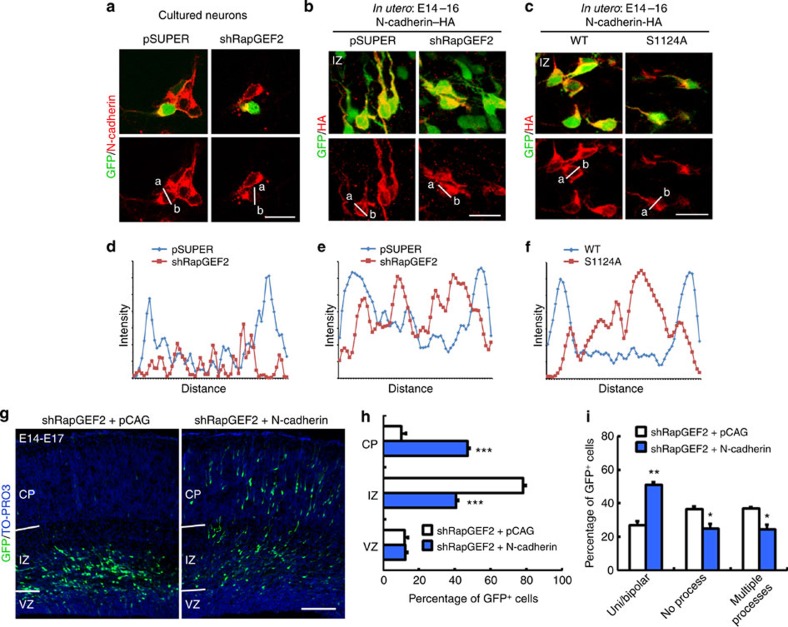
RapGEF2 phosphorylation regulates N-cadherin function. (**a**) Immunostaining of GFP and endogenous N-cadherin in cultured cortical neurons. E14 mouse brains were co-electroporated *in utero* with vector control (pSUPER) or RapGEF2 shRNA (shRapGEF2) together with GFP plasmid. Cortical neurons from E16 electroporated cortex were cultured *in vitro* for 2 days and analysed by immunocytochemistry. (**b**) E16 brain sections electroporated with vector control (pSUPER) or RapGEF2 shRNA (shRapGEF2) together with pCAG-based N-cadherin probe (N-cadherin–HA) were immunostained for GFP and HA. (**c**) E16 brain sections electroporated with WT or phosphodeficient mutant (S1124A) of RapGEF2 plasmids together with N-cadherin–HA at E14 were immunostained for GFP and HA. Scale bars, 20 μm. The experiments were repeated for at least three times. (**d**–**f**) Quantification of the fluorescence intensity profiles of endogenous (**d**) and ectopically expressed (**e**,**f**) N-cadherin across the cell bodies of transfected neurons in the intermediate zone. The red fluorescence intensity across the representative neurons from *a* to *b* as indicated in the panels above was measured by ImageJ software. (**g**) Co-electroporation of E14 mouse brains with vector control (pCAG) or pCAG-based N-cadherin-expressing plasmid together with RapGEF2 shRNA (shRapGEF2) was performed. Representative E17 coronal cortices were stained for GFP and TO-PRO3. Scale bar, 100 μm. The experiment was repeated for at least three times. (**h**) Quantification of the percentages of GFP^+^ neurons in different cortical layers. Error bars indicate the s.e.m. of five different brains containing >800 neurons. (**i**) Quantification of the percentages of neurons with uni- or bipolar morphology, no process and multiple (≥3) processes. Error bars indicate the s.e.m. of three different brains containing >120 neurons. *P<0.05, **P<0.01, ***P<0.001 versus shRapGEF2+pCAG group; Student’s *t*-test.

RapGEF2 functions as an important GEF for Rap1 activation. In many cell types, Rap1 regulates the trafficking of cell surface molecules between endosomes and the plasma membrane^35^,^36^. Interestingly, recent evidence suggests that Rap1 regulates N-cadherin localization in cortical neurons during neuronal migration^18^. Accordingly, to determine whether the subcellular localization of endogenous N-cadherin is regulated by RapGEF2, E14 mouse brains were electroporated with pSUPER or RapGEF2 shRNA. GFP^+^ regions were resected from the transfected cortices, and cortical neurons were cultured *in vitro*. Neurons were examined by immunocytochemical analysis 2 days later. Although endogenous N-cadherin localized at the cell membrane in control neurons, the protein was predominantly localized in the perinuclear region in RapGEF2-depleted neurons (Fig. 8a,d). To determine whether RapGEF2 regulates the membrane localization of N-cadherin *in vivo*, C-terminal hemagglutinin (HA)-tagged full-length N-cadherin (N-cadherin–HA) was introduced together with pSUPER or RapGEF2 shRNA into E14 mouse brains by *in utero* electroporation. The brains were analysed 2 days after electroporation, when most of the neurons were in the intermediate zone and presumably undergoing multipolar–bipolar transition. N-cadherin–HA was present on the cell membrane, internal compartments and cell processes under both conditions; however, the relative amount of N-cadherin–HA on the membrane of neurons was lower under RapGEF2 suppression (Fig. 8b,e). To elucidate whether N-cadherin distribution is specifically regulated by Cdk5-mediated RapGEF2 phosphorylation at Ser1124, RapGEF2 (WT) or S1124A mutants were co-electroporated with pcDNA3–N-cadherin–HA in E14 mouse brains. The cell surface N-cadherin–HA decreased with RapGEF2 S1124A co-expression when compared with that of RapGEF2 (Fig. 8c,f). In addition, Cdk5 loss of function altered the protein level and spatial expression pattern of N-cadherin. At E15, N-cadherin was strongly expressed in the intermediate zone of wild-type cortices, whereas its protein level was much lower in the intermediate zone of Cdk5-deficient cortices (Supplementary Fig. 9a). These results collectively suggest that RapGEF2 and Cdk5-dependent RapGEF2 phosphorylation are involved in the regulation of N-cadherin surface localization in migrating neurons.

N-cadherin has been suggested to orient multipolar neurons towards a bipolar morphology under the control of Rap1 (ref. 18). Therefore, we investigated whether RapGEF2 regulates neuronal migration by modulating N-cadherin. Moderate N-cadherin expression in RapGEF2-suppressed cortices significantly rescued the defective bipolar transition and neuronal migration (Fig. 8g–i). Furthermore, migrating bipolar neurons with strong phospho-RapGEF2 signals were associated with nestin-labelled radial glial scaffold (Supplementary Fig. 9b). Our data collectively suggest that Cdk5/RapGEF2 signalling acts upstream of Rap1/N-cadherin in the upper intermediate zone to promote multipolar–bipolar transition and proper neuronal entry into the cortical plate, presumably through cell adhesion between migrating neurons and radial glial fibres.

## Discussion

Cerebral cortex development is vulnerable to disruption during the establishment of polarity in multipolar migrating neurons in the intermediate zone^2^,^4^. However, the detailed mechanism underlying the regulation of leading process specification remains elusive. The present study provides evidence that the dynamic expression of RapGEF2 corresponds to the timing of polarity acquisition during neuronal migration. RapGEF2 specifically governs the multipolar–bipolar transition via Rap1/N-cadherin signalling, thereby specifying the polarity and safeguarding the proper assembly of pyramidal precursors into the cerebral cortex. The small GTPase Rap1 acts as the immediate signal transducer to translate the action of RapGEF2 into functional output via N-cadherin. Intriguingly, the phosphorylation of RapGEF2 by Cdk5 regulates the GEF activity of RapGEF2 and facilitates Rap1 activation during multipolar–bipolar transition; thus, this phosphorylation event functions as an essential molecular switch for neuronal migration. Taken together, we provide mechanistic insights into the functional roles of RapGEF2 in neuronal migration.

In addition to locomotion and somal translocation, multipolar migration has been recently recognized as the third mode of neuronal migration in the developing cerebral cortex^2^,^4^. Multipolar pyramidal precursors undergo unidirectional movement in the lower intermediate zone, and eventually transit into bipolar morphology to resume radial migration as they move to the upper intermediate zone. Although several molecules, such as filamin A, LIS1 and DCX, have been proposed to regulate multipolar–bipolar transition^4^, their pervasive expression in the entire intermediate zone cannot explain how the transition is initiated and regulated. Importantly, our results revealed a specific expression pattern of RapGEF2 with prominent upregulation in the upper intermediate zone that precisely corresponds to the timing of bipolar transition. RapGEF2 is the first protein preferentially expressed in neurons in the upper intermediate zone and not in the lower intermediate zone that has been identified to date. The only other similar protein is FoxG1, although it is expressed in radial glial cells in addition to migrating neurons^37^. Furthermore, we have provided conceptual advances in the understanding of the regulation of Cdk5 activity in the developing cortex. While Cdk5 is critical for neural development, its activity was suggested to be ubiquitous in postmitotic neurons^9^. We present the first demonstration that Cdk5 is preferentially activated in radially migrating neurons during cortical development as evidenced by the restricted expression of p35 in the radial migration zone. Thus, besides the dynamic expression of RapGEF2, Cdk5-dependent phosphorylation provides another level of control of RapGEF2 activity to ensure proper neuronal migration and cortical circuit formation.

The coordinated modes of neuronal migration are controlled by both extrinsic signals and intrinsic developmental programs. Reelin is a well-documented extracellular cue required for neuronal migration. Recent studies suggest that the Reelin/Dab1 pathway is primarily required for somal translocation but not glia-guided locomotion in the developing mouse cortex^17^. Furthermore, the action of Reelin on migrating neurons is dependent on C3G and Rap1, which facilitates translocation by stabilizing leading processes towards the marginal zone^17^,^19^. Cdk5 is a major intrinsic regulator of neuronal migration. Recent evidence suggests that Cdk5 is mainly involved in multipolar–bipolar transition but also regulates glia-guided locomotion via leading process dynamics and nucleokinesis^15^. Nonetheless, how the extracellular cues and intracellular programs are integrated and coordinated to control distinct neuronal migration modes remains largely enigmatic. Rap1 modulates multiple steps of neuronal migration, including multipolar–bipolar transition and somal translocation; thus, it plays multi-faceted roles in neuronal migration^17^,^18^,^19^. Accordingly, Rap1 may be differentially regulated by specific GEFs in a spatiotemporal-specific manner, subsequently triggering the downstream signalling pathways essential for distinct migration modes. The present results show that the RapGEF2-dependent activation of Rap1 functions in multipolar–bipolar transition and neuronal entry into the cortical plate, but not in somal translocation. Although the C3G-dependent activation of Rap1 is dispensable for the bipolar transition, it appears to be important for the later migration phase in the cortical plate, particularly terminal translocation. Thus, while Rap1 is involved in different cellular events during neuronal migration, its activity may be controlled by different GEFs, which is consistent with findings in genetic mouse models^25^,^38^. Collectively, these results suggest that multiple signalling pathways may converge at Rap1 via the activation of specific GEFs at different stages of neuronal migration, that is, Cdk5-activated RapGEF2 for multipolar–bipolar transition and Reelin-activated C3G for somal translocation^17^,^19^.

Given the Cdk5-dependent regulation of RapGEF2 activity and Reelin-mediated C3G activation^39^, we propose a sequential regulation model of the relationship between the two major neuronal migration pathways: the Cdk5- and Reelin-dependent pathways. In brief, these two pathways are not simply in parallel, but rather act on successive neuronal migration phases. Disruption of the previous migration phase results in a failure to unlock responsiveness to signalling molecules that initiate subsequent migration phases. Accordingly, Cdk5-mediated RapGEF2 phosphorylation mainly controls multipolar–bipolar transition as a cellular checkpoint and allows bipolar locomotion in the cortical plate as well as the subsequent terminal translocation activated through the Reelin-mediated C3G pathway.

The proper migration of cortical pyramidal neurons in the developing neocortex requires precisely orchestrated events including cytoskeletal rearrangement and cell adhesion^6^,^38^. Numerous studies have characterized Cdk5 substrates that modulate cytoskeletal reorganization^40^; however, how cell adhesion is regulated by Cdk5 during neuronal migration remains unclear. Notably, it has been suggested that N-cadherin can be regulated by Cdk5 in cultured cortical neurons via β-catenin phosphorylation^41^. We and others demonstrated that N-cadherin-mediated adhesion is required for neuronal migration, although excessive surface N-cadherin inhibits migration^17^,^18^,^37^. Cdk5-mediated RapGEF2 phosphorylation modulates the surface localization of N-cadherin in migrating neurons in the intermediate zone, and proper N-cadherin expression level rescues the multipolar–bipolar transition and migration of RapGEF2-suppressed neurons. Taken together, surface N-cadherin levels are likely to be tightly controlled by Cdk5-mediated signalling and other pathways^37^ during cortical development. In particular, Cdk5-dependent RapGEF2 phosphorylation is specifically required for multipolar–bipolar transition via the control of N-cadherin-mediated adhesion.

Rap1 plays multi-faceted roles in an array of neurodevelopmental processes, including neuronal migration^17^,^18^,^19^, dendrite development^42^ and spine morphogenesis^43^. Thus, it is not surprising that disease-associated genetic variants of Rap1 GEFs have been identified, which are often linked to Rap1 deregulation and neurodevelopmental deficits. For instance, overexpression of the autism-associated variants of RapGEF4 (also known as Epac2) leads to impaired development of basal dendrites and altered spine morphology in cultured cortical and hippocampal neurons^44^. Intriguingly, copy number variants of RapGEF2 and RapGEF6 (a close homologue of RapGEF2) have been identified in schizophrenia patients^45^,^46^. Therefore, it is of interest to further characterize the physiological functions of the Cdk5-dependent regulation of Rap1 signalling via RapGEF2 and RapGEF6 in psychiatric disorders such as schizophrenia.

In conclusion, the present study indicates that the dynamic regulation of RapGEF2 activity in migrating neurons is critical for their integration into the cerebral cortex. Our results demonstrate that the Cdk5-dependent activation of RapGEF2, spatial activation of Rap1 signalling and Rap1-facilitated surface localization of N-cadherin in the upper intermediate zone control neuronal migration and ultimately the architecture of the mammalian cerebral cortex.

## Methods

### Plasmids and antibodies

The shRNA target sequences for RapGEF2, C3G or Cdk5 are as follows: shRapGEF2: 5′- GAGAGATTGTAATGGTGAA -3′ (ref. 26), shRapGEF2-2: 5′- GTCATTAACCAGGAAACAT -3′, shC3G: 5′- GGACTTTGATGTTGAATGT -3′ (ref. 31) and shCdk5: 5′- CCGGGAGATCTGTCTACTC -3′. Two shRNAs for RapGEF2 recognize both rat and mouse versions of RapGEF2 sequence, while shRapGEF2 was primarily used throughout the experiments. The scrambled shRNA sequence for shRapGEF2 is 5′- AATTGTAATAGGATGGGAG -3′. The shRNAs and scrambled shRNA were cloned into SUPER vector. Expression constructs containing the sequences of full length or fragment (RapGEF2-ct: amino acids 957–1,266) of RapGEF2 (Kazusa DNA Research Institute, KIAA0313)^47^, Rap1 (Invitrogen) and N-cadherin (Addgene plasmid 18870)^48^ were generated by PCR and inserting the corresponding coding region into the pcDNA3 vector or pCAGIG vector^49^. Indicated mutants of FLAG-RapGEF2-ct, the shRNA-resistant form, and phosphomutants of RapGEF2 as well as the constitutive active mutant of Rap1 (63E) were generated using the QuikChange II XL Site-Directed Mutagenesis Kit (Stratagene). Expression constructs encoding human Cdk5 and mouse p35 have been described previously^50^. All plasmids were prepared in endotoxin-free conditions using QIAGEN Plasmid Maxi Kit (Qiagen).

The antibodies used for immunofluorescence and western blot analysis are listed below. Monoclonal antibodies: mouse anti-GFP (1:1,000, A11120, http://ctru.org/861101) and anti-N-cadherin (1:200, 333900, http://ctru.org/861102) from Invitrogen; mouse anti-FLAG (1:5,000, F1804, http://ctru.org/861103), anti-Tuj1 (1:500, T8660, http://ctru.org/861104), anti-CS-56 (1:500, C8035, http://ctru.org/861105) and anti-β-actin (1:4,000, 3853, http://ctru.org/861107) from Sigma; mouse anti-Cdk5 (DC-17; 1:2,000, sc-249, http://ctru.org/861108) from Santa Cruz Biotechnology; rabbit anti-p35 (C64B10; 1:500, 2680, http://ctru.org/861109) and anti-cleaved caspase-3 (1:200, 9661, http://ctru.org/861110) from Cell Signaling Technology; and mouse anti-nestin (rat-401; 1:500, MAB353, http://ctru.org/861111) from Millipore. Polyclonal antibodies: rabbit anti-HA (1:500, sc-805, http://ctru.org/861112) and anti-Cux1 (1:500, sc-13024, http://ctru.org/861113) from Santa Cruz Biotechnology; rabbit anti-Pax6 (1:500, AB2237, http://ctru.org/861114) from Millipore; anti-Tbr2 (1:500, ab23345, http://ctru.org/861115) and rat anti-Ctip2 (1:500, ab18465, http://ctru.org/861116) from Abcam; rabbit anti-GFP (1:2,000, 598, http://ctru.org/861117) from MBL; and rabbit anti-RapGEF2 (1:1,000, A301-966A, http://ctru.org/861118) from Bethyl Laboratories. Goat secondary antibodies conjugated with Alexa Fluor 488 (1:2,000, http://ctru.org/861119) and 546 (1:2,000, http://ctru.org/861120) were purchased from Molecular Probes.

The phospho-specific antibody against phosphorylated RapGEF2 at Ser1124 (p-RapGEF2, 1:500, http://ctru.org/861106) was generated by immunizing a rabbit with a synthetic phosphopeptide containing the phospho-Ser1124 residue of RapGEF2 (SPQSS(P)PRKGYTLAC, amino acids 1,120–1,132; Bio-Synthesis). The serum from the immunized rabbit was first purified using a nonphosphorylated peptide (PTSPQSSPRKGYTLC; Bio-Synthesis) coupled to a Sulfolink column (Pierce Biotechnology), whose elution resulted in the total RapGEF2 antibody (1:500, http://ctru.org/861107). The phospho-specific antibody was then purified by another Sulfolink column conjugated with the phosphopeptide.

### *In utero* electroporation and tissue processing

Cdk5- and p35-knockout mice were described previously^50^. Institute of Cancer Research (ICR) mouse embryos of either sex were randomly selected and electroporated *in utero* as described previously^49^. Briefly, the uteri of E12 or E14 pregnant mice anaesthetized with pentobarbital were exposed and placed on humidified gauze pads. Plasmids mixed with 0.05% Fast Green (1 μl; Sigma) were injected into the lateral ventricle of the recipient embryos. Indicated plasmids were mixed at the following concentrations: shRNA plasmids, 1 μg μl^−1^; pCAGIG vector, 1 μg μl^−1^; shRNA-resistant rescue plasmids, 4 μg μl^−1^; and pCAGIG-Rap1 CA, pCAGIG–N-cadherin–HA and pcDNA3–N-cadherin–HA, 1 μg μl^−1^. Immediately after DNA injection, five 50-ms electrical pulses (28 V for E12 and 33 V for E14) were applied at 1-s intervals using a 5-mm electrode (CUY21E, Nepagene) and an electroporator (EM830, BTX) to electroporate plasmids into the lateral cortex (if not specified elsewhere). The uterine horns were returned to the abdominal cavity, and the abdomen wall and skin were quickly sutured. All surgical procedures were completed within 30 min, after which the mice recovered on a heating plate until waking up. Mice were housed in a vivarium (one pregnant mouse per cage) with a 12/12-h light/dark cycle. Experiments using animals were conducted in accordance with the guidelines approved by the Animal Care Committee at the Hong Kong University of Science and Technology.

The embryos or mice were killed at the indicated stages (E15, E17, P2 or P20) and subjected to cardiac perfusion with 4% paraformaldehyde (PFA; wt/vol) in phosphate-buffered saline (PBS). After perfusion, the forebrains were removed and post-fixed in 4% PFA–PBS solution (wt/vol) for another 2 h and then changed to 30% sucrose PBS solution (wt/vol) overnight at 4 °C. The brains were embedded and frozen in Optimal Cutting Temperature (OCT) compound before being cut into 20-μm coronal sections using a cryostat (HM 560, Microm). For the mice examined at P20, the perfused cortices were fixed in 4% PFA–PBS solution (wt/vol) for 24–48 h. Coronal sections (80-μm thick) were prepared by a vibratome (Leica).

### Cell cultures and transfection

HEK293T cells were fed in Dulbecco’s modified Eagle medium (Invitrogen) with 10% heat-inactivated fetal bovine serum (GIBCO-BRL; vol/vol) plus antibiotics. HEK293T cells were transfected using Lipofectamine with Plus Reagent (Invitrogen) according to the manufacturer’s instructions. Cells were collected 24 h after transfection for immunoprecipitation and western blot analysis.

Primary cortical neurons were collected from rat brains at E18 and maintained as described previously^51^. Briefly, dissociated cortical neurons were seeded on poly-D-lysine (Sigma)-coated petri dishes and cultured in Neurobasal medium (Invitrogen) containing 2% B27 (Invitrogen; vol/vol) at 37 °C with 5% CO_2_. Cortical neurons at 0 day *in vitro* were transfected with indicated plasmids using the Amaxa Nucleofector (Lonza AG). Western blot analysis was performed at 3 days *in vitro* after seeding. To examine the knockdown efficiency of RapGEF2 *in vivo*, primary cortical neurons were collected from E16 mouse brains electroporated with pSUPER vector or shRapGEF2 at E14.

### Immunofluorescence and image acquisition

For immunocytochemistry, dissociated neurons were fixed in 4% PFA–PBS solution (wt/vol) for 20 min at room temperature 25 °C. Fixed neurons were permeabilized and blocked in PBS containing 1% bovine serum albumin (wt/vol), 4% goat serum (vol/vol) and 0.4% Triton X-100 (vol/vol) for 30 min at room temperature 25 °C. Coverslips were stained with indicated primary antibodies overnight at 4 °C followed by corresponding fluorescence-conjugated secondary antibodies for 1 h at 25 °C. For immunohistochemistry, all reactions were performed in a Tris-buffered saline with Triton X-100 (TBST) solution containing 0.1% Triton X-100 (vol/vol) and 3% bovine serum albumin (wt/vol). The slides were permeabilized and blocked in TBST solution for 30 min at room temperature 25 °C and subsequently incubated with indicated primary antibodies overnight at 4 °C. Next, the slides were washed in TBST solution for 20 min and immunostained with corresponding secondary antibodies plus TO-PRO3 (Invitrogen) for 1 h at room temperature 25 °C. Finally, the slides were mounted in Mowiol mounting medium.

Representative Z-series confocal images of cortical sections were acquired at a 1-μm step with 10–12 optical sections using Olympus Fluoview FV1000 confocal microscope with a × 10, × 20 or × 40 objective. Images of individual cultured neurons were collected at a single layer with a × 40 objective.

### Live-imaging analysis

Live imaging of neuronal migration in the mouse cortical slice cultures was performed as described^51^. *In utero* electroporation was performed at E14. At E16, the electroporated brains were rapidly dissected and cut into 300-μm coronal slices using a vibratome (Leica). Cortical slices were then grown in Neurobasal medium (Invitrogen) containing 2% B27 (Invitrogen; vol/vol) at 37 °C with 5% CO_2_. Time-lapse images were acquired every 15 min for 13 h (× 10 objective) or 7 h (× 40 objective) using a confocal microscope (Leica SP8).

### *In vitro* phosphorylation assay and Rap1 pull-down assay

HEK293T cells, primary neurons or brain tissues were homogenized in lysis buffer (50 mM Tris (pH 8.0), 150 mM NaCl, 2 mM EDTA, 50 mM NaF, 1 mM dithiothreitol, 0.25% sodium deoxycholate, 0.5% Nonidet P-40 and 10% glycerol) supplemented with protease inhibitor cocktail.

For the *in vitro* phosphorylation assay, the indicated constructs were overexpressed in HEK293T cells. FLAG-tagged WT or phosphomutants of RapGEF2 or RapGEF2-ct were immunoprecipitated from 293T cell lysates using anti-FLAG M2 agarose beads (Sigma) and incubated with recombinant Cdk5/p35 for 30 min at 30 °C in 50 μl kinase buffer. The samples were resolved by SDS–PAGE followed by either western blot analysis with specific antibodies or autoradiography.

To measure the activity of Rap1, a GST-RalGDS-RBD pull-down of Rap1-GTP from cell or brain lysates followed by Rap1 western blot analysis was performed according to the manufacturer’s instructions (Active Rap1 Pull-Down and Detection Kit, Thermo Scientific, #89872).

### Quantitative analysis

The significance of differences between two groups was examined using unpaired Student’s *t*-tests or one-way analysis of variance with *post hoc* Newman–Keuls test across *n* samples, where *n* is the number of brains or cells as indicated in the figure legends. All data are presented as mean±s.e.m. unless specified otherwise. No data points were excluded. The intensity grey value of a line drawn across the cell body (vertical to the primary neurite) of a transfected neuron from *a* to *b* was determined using ImageJ (NIH, USA).

## Author contributions

N.Y.I. supervised the project. T.Y., A.K.Y.F. and N.Y.I. designed the study, analysed the data and wrote the manuscript. T.Y. and J.P.K.I. performed the experiments. All authors discussed the experimental results and commented on the manuscript.

## Additional information

**How to cite this article:** Ye, T. *et al*. Cdk5-mediated phosphorylation of RapGEF2 controls neuronal migration in the developing cerebral cortex. *Nat. Commun.* 5:4826 doi: 10.1038/ncomms5826 (2014).

## Supplementary Material

Supplementary Figures Supplementary Figures 1-10

Supplementary Movie 1 This movie shows the migration of neurons following co-electroporation of pSUPER vector and GFP plasmids. Most of the neurons attained bipolar morphology, underwent nuclear translocation and migrated to the cortical plate. Images were captured every 15 min for 13 h.

Supplementary Movie 2 This movie shows the migration of neurons following co-electroporation of RapGEF2 shRNA and GFP plasmids. RapGEF2 deficient neurons were unable to enter into the cortical plate, and showed local movement within the intermediate zone. Images were captured every 15 min for 13 h.

Supplementary Movie 3 This movie shows an example of migrating neurons within the IZ following co-electroporation of pSUPER vector and GFP plasmids. The neuron exhibited active process extension and retraction in random directions, and eventually formed a radially-oriented leading process. Images were captured every 15 min for 7 h.

Supplementary Movie 4 This movie shows an example of migrating neurons within the IZ following co-electroporation of RapGEF2 shRNA and GFP plasmids. The RapGEF2 deficient neuron exhibited active process extension and retraction in random directions, but failed to form a radially-oriented leading process. Images were captured every 15 min for 7 h.
