# Supplementary material for: Cdk5-mediated phosphorylation of RapGEF2 controls neuronal migration in the developing cerebral cortex
Source: Nat Commun. 2014 Sep 5;5:4826. doi: 10.1038/ncomms5826 (PMC4164783; doi:10.1038/ncomms5826)
Supplement: Supplementary Figures — 1-10 [file ncomms5826-s1.pdf]

Supplementary Figure 1

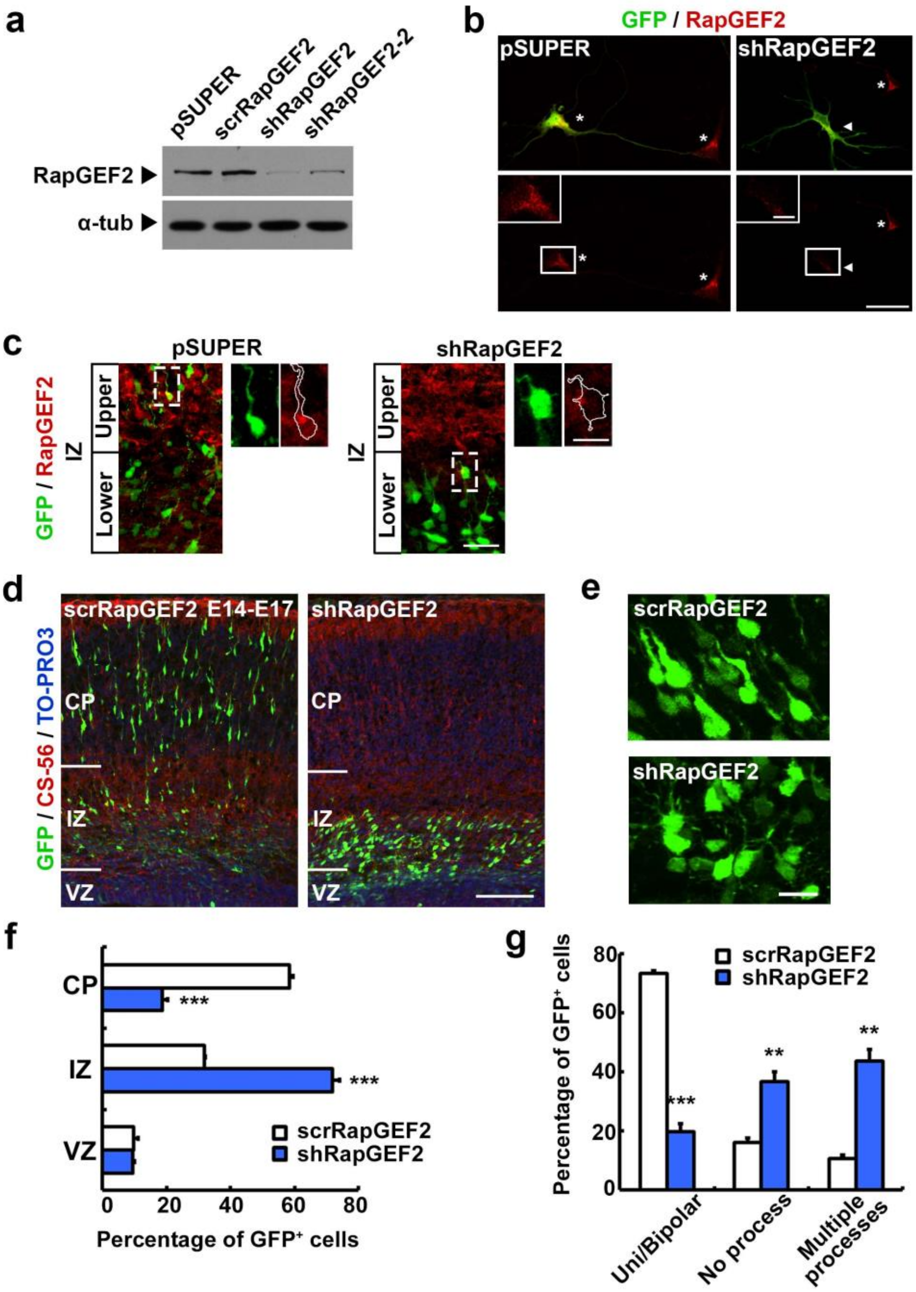

Supplementary Figure 1 RapGEF2 is essential for neuronal migration in the neocortex. (a) Knockdown efficiency of shRNAs for RapGEF2 shown by western blot analysis. pSUPER vector (pSUPER), scrambled shRNA (scrRapGEF2), and indicated shRNAs (shRapGEF2 and shRapGEF2-2) were transfected into primary cultured cortical neurons. Proteins were harvested on 3 DIV, followed by blotting using anti-RapGEF2 antibody. α-tub served as the loading control. (b) Validation of knockdown efficiency of RapGEF2 shRNA by immunocytochemical analysis. Primary cultured cortical neurons were set using E16 mouse cortices after electroporation of indicated shRNA constructs with GFP-expressing vector at E14. Neurons were fixed and stained for GFP and RapGEF2. Scale bars: 5 and 20 µm. (c) Validation of knockdown efficiency of RapGEF2 shRNA by immunohistochemical analysis *in vivo*. E14 mouse brains were co-electroporated with the control (pSUPER) or RapGEF2 shRNA (shRapGEF2) together with GFP plasmid. E17 cortical sections were stained for GFP and RapGEF2. Scale bars: 25 and 10 µm. (d) E14 mouse brains were co-electroporated with scrambled shRNA (scrRapGEF2) or RapGEF2 shRNA (shRapGEF2) together with GFP plasmid. Representative E17 cortical sections were stained for GFP, CS-56 (a subplate marker), and TO-PRO3. Scale bar: 100 µm. At least 3 different brains for each group were examined. (e) Magnified images of electroporated neurons in the intermediated zone. Scale bar: 20 µm. (f) The percentages of GFP^+ neurons in different cortical layers were quantified. Error bars indicate the SEM of 5 different brains containing >800 neurons. (g) The percentages of neurons with uni- or bipolar morphology, no process, and multiple (≥3) processes were quantified. Error bars indicate the SEM of 3 different brains containing >120 neurons. $^{**}P < 0.01$, $^{***}P < 0.001$ versus scrRapGEF2 group; Student's *t*-test.

Supplementary Figure 2

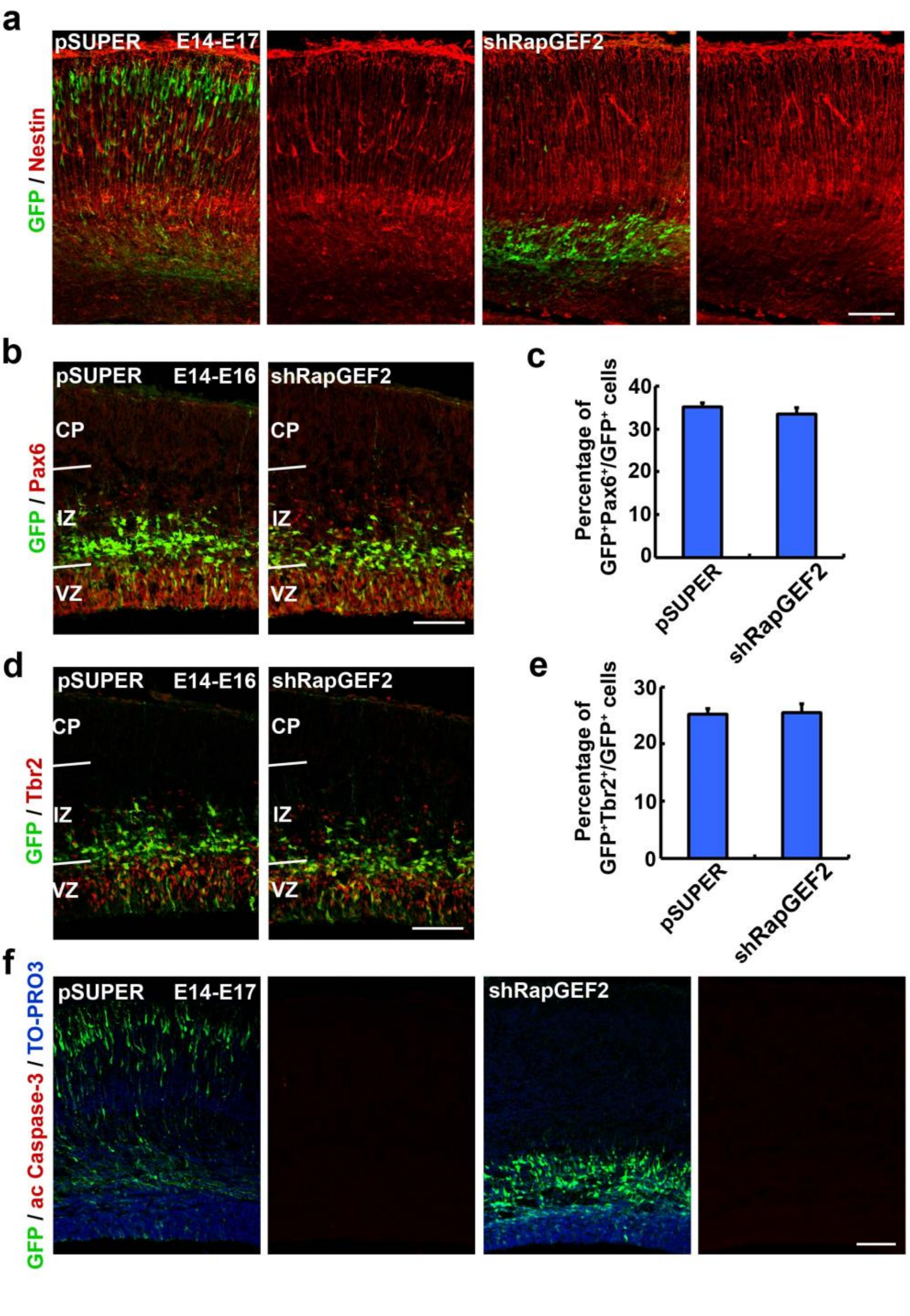

Supplementary Figure 2 RapGEF2 does not affect radial glia organization, **apical or basal progenitor maintenance**, **or neuronal survival.** (a) Representative coronal brain sections of E17 mouse embryos electroporated with indicated plasmids at E14 were stained for GFP and Nestin, a marker for radial glial fibers. (b, d) Representative coronal sections from E16 mouse brains electroporated with the indicated plasmids at E14 were stained for GFP and the apical progenitor marker Pax6 (b) or intermediate progenitor marker Tbr2 (d) together with nuclear marker TO-PRO3. (c, e) Quantification of the percentages of $Pax6^+$ and $Tbr2^+$ neural progenitors in GFP^+ cells revealed no significant difference between control and RapGEF2-depleted brains. Error bars indicate the SEM of 3 different brains containing >600 neurons. (f) *In utero* electroporation of E14 mouse brains was performed using vector control (pSUPER) or RapGEF2 shRNA (shRapGEF2) together with GFP plasmid. Representative E17 coronal sections from E17 mouse brains electroporated with pSUPER or shRNA were stained for GFP, apoptosis marker cleaved caspase-3 (ac caspase-3), and nuclear marker TO-PRO3. Scale bars: 100 µm (a, b, d, f). At least 3 different brains for each group were examined.

Supplementary Figure 3

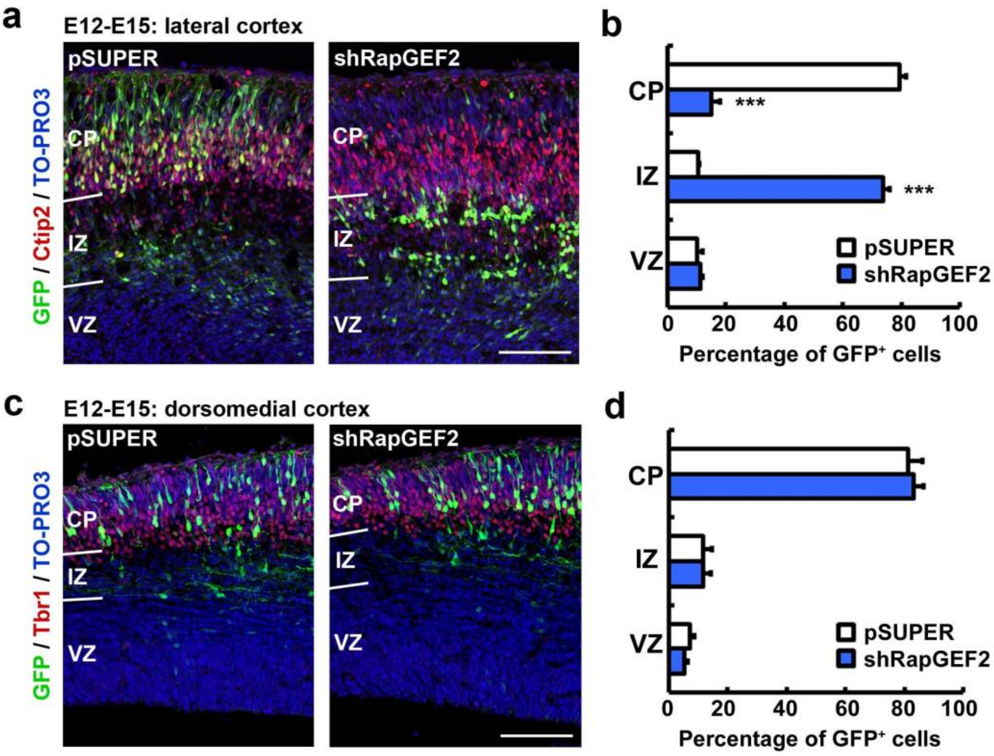

Supplementary Figure 3 RapGEF2 is required in early-born neurons for multipolar–bipolar transition and onset of locomotion, but not for somal translocation. (a, c) pSUPER vector (pSUPER) or RapGEF2 shRNA (shRapGEF2) together with GFP plasmid was electroporated into the lateral cortex (a) or dorsomedial cortex (c) of E12 mouse brains. Three days after electroporation, E15 brain sections were collected and stained for GFP, layer V neuron marker Ctip2, and nuclear marker TO-PRO3. Scale bar: 100 µm. (b, d) The percentages of GFP^+ neurons in different cortical layers were quantified. Error bars indicate the SEM of 3 different brains containing >600 neurons. ***$P < 0.001$; Student's *t*-test.

Supplementary Figure 4

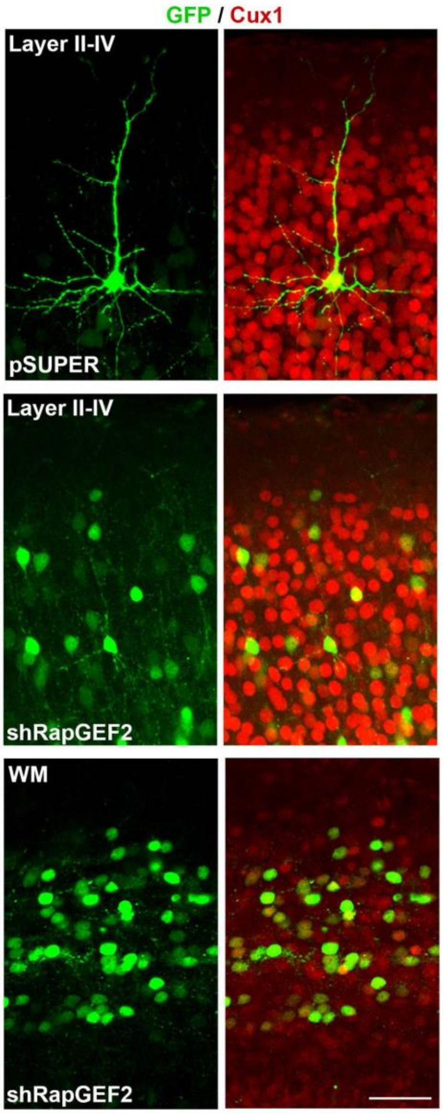

Supplementary Figure 4 RapGEF2-suppressed neurons exhibit disrupted morphology at P20. *In utero* electroporation of E14 mouse brains was performed using pSUPER vector (pSUPER) or RapGEF2 shRNA (shRapGEF2). Coronal cortical sections from P20 mouse cortices were stained for GFP (green) and Cux1 (a layer II–IV marker). Representative images of pSUPER-electroporated neurons in layer II–IV and RapGEF2-suppressed neurons, which were stained positive for Cux1 in the corresponding layers and white matter, are shown at higher magnification. Scale bar: 50 µm. At least 3 different brains for each group were examined.

Supplementary Figure 5

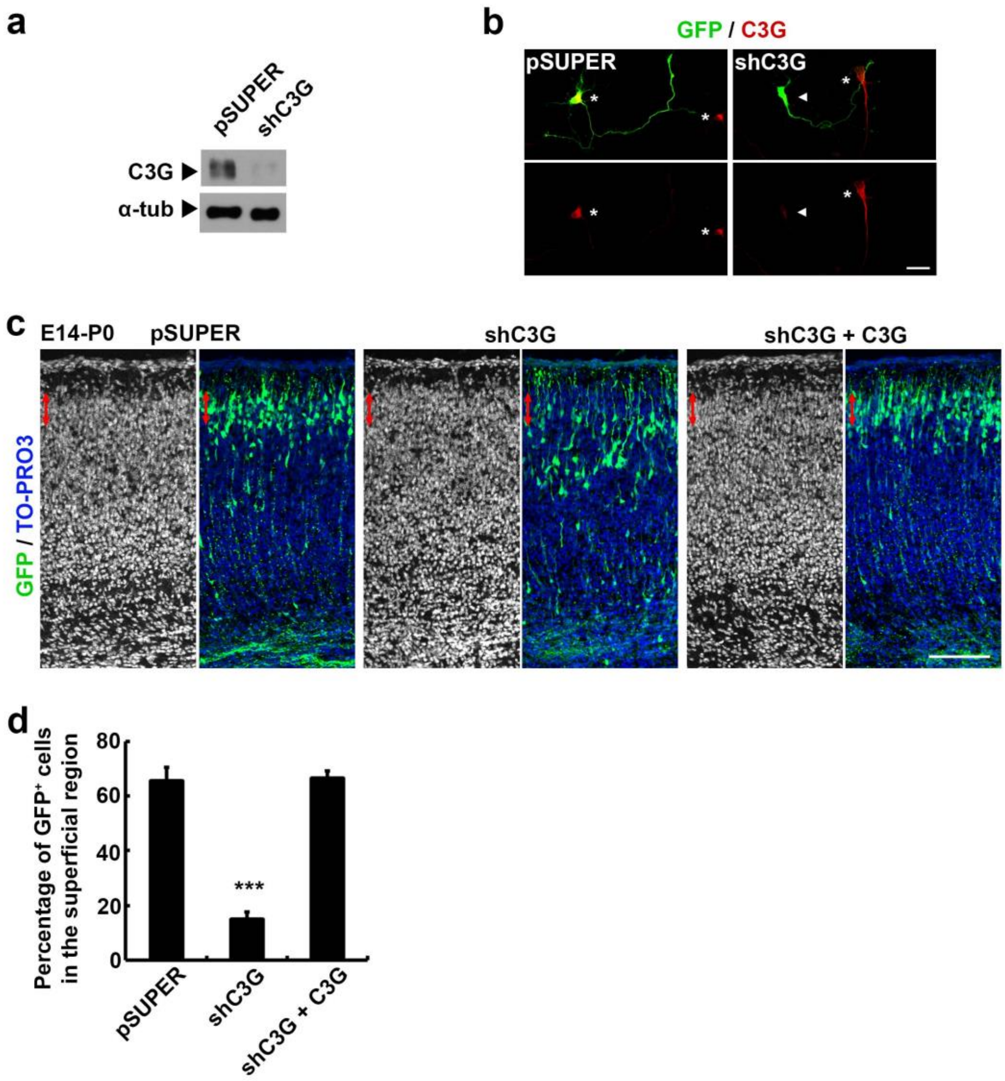

Supplementary Figure 5 C3G is not required for the multipolar–bipolar transition of newborn neurons and neuronal entry into the cortical plate. (a) Knockdown efficiency of shRNA for C3G shown by western blot analysis. pSUPER vector (pSUPER) and C3G shRNA (shC3G) were transfected into primary cultured cortical neurons. Proteins were harvested on 3 DIV, followed by blotting using anti-C3G antibody. α-tub served as the loading control. (b) Validation of knockdown efficiency of C3G shRNA by immunocytochemical analysis. Primary cultured cortical neurons were set using E16 mouse cortices after electroporation of indicated shRNA constructs with GFP-expressing vector at E14. Neurons were fixed and stained for GFP and RapGEF2. Scale bar: 20 µm. (c) Co-electroporation of E14 mouse brains was performed using pSUPER or shC3G together with GFP plasmid, or shC3G plus a non-targetable C3G-expressing plasmid. E17 coronal cortical sections were stained for GFP and TO-PRO3. Scale bar: 100 µm. (f) Quantification of the percentages of GFP^+ cells migrating into the superficial region of the cerebral cortex at P0, indicated by the double-headed arrows. Error bars indicate the SEM of 3 different brains containing >500 neurons.

Supplementary Figure 6

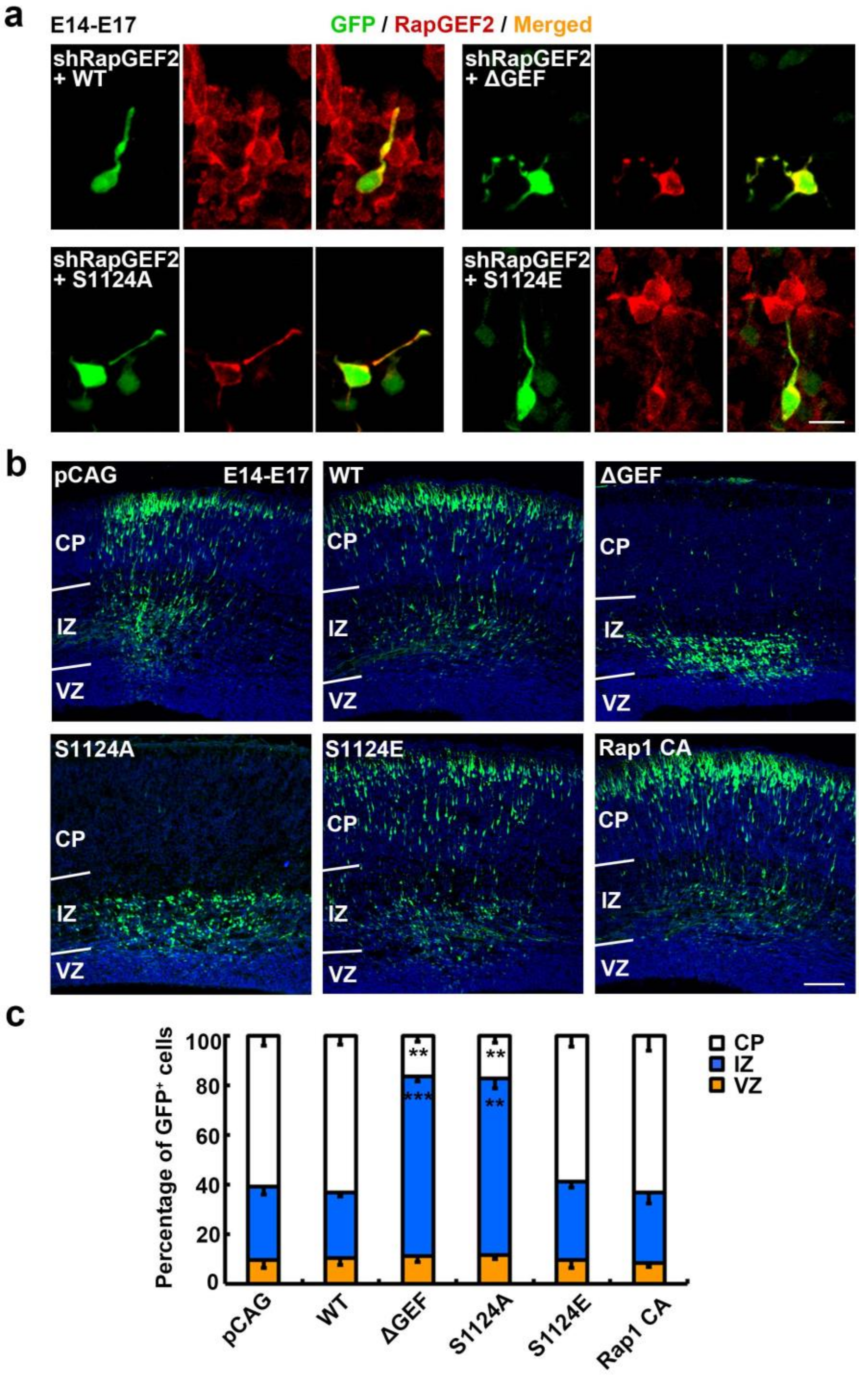

Supplementary Figure 6 Effects of RapGEF2 overexpression in neuronal migration. (a) Validation of RapGEF2 expression in rescue experiments. RapGEF2 was mainly localized in cytoplasm instead of nucleus *in vivo*. GFP^+ neurons co-expressed with wild-type or mutant RapGEF2 were confirmed by staining for GFP and RapGEF2. Scale bar: 20 μm. (b) E14 mouse brains were electroporated with indicated plasmids, followed by distribution analyses of GFP^+ neurons at E17. Scale bar: 100 μm. (c) The percentages of GFP^+ neurons in different cortical layers were quantified. Error bars indicate the SEM of 3 different brains containing >600 neurons. **$P < 0.01$, ***$P < 0.001$ versus pSUPER; Student's *t*-test.

Supplementary Figure 7

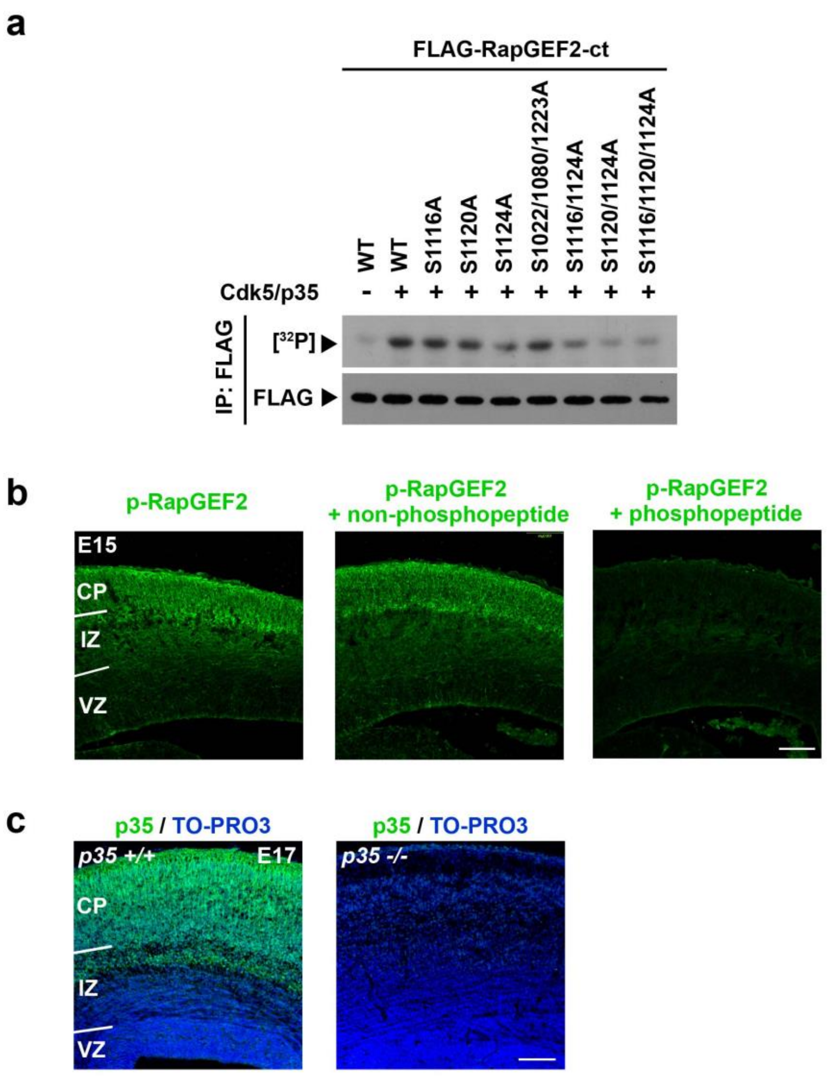

Supplementary Figure 7 Cdk5 phosphorylates RapGEF2 at Ser1124. (a) Wild-type (WT) or indicated mutant forms of RapGEF2-ct (a.a. 957–1266) were expressed in HEK293T cells followed by immunoprecipitation and *in vitro* kinase assay without or with Cdk5/p35. (b) Specificity validation of custom antibody against phospho-RapGEF2 antibody (p-RapGEF2) in the mouse neocortex. E15 coronal cortices were immunostained with p-RapGEF2, or p-RapGEF2 pre-incubated with non-phosphopeptide or phospho peptide. (c) Specificity validation of anti-p35 antibody in the mouse neocortex. Wild-type or p35-deficient cortical sections at E17 were stained for p35 and TO-PRO3. Scale bars: 100 μm (b, c).

Supplementary Figure 8

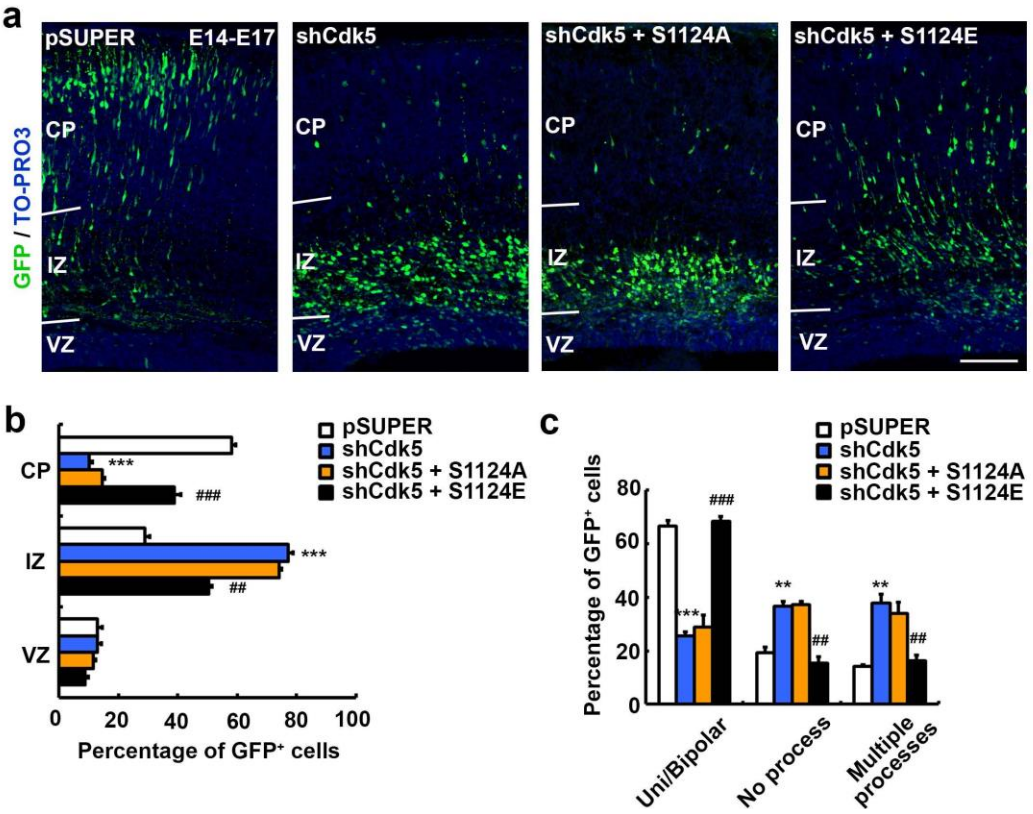

Supplementary Figure 8 RapGEF2 phosphorylation at Ser1124 is important for Cdk5-dependent neuronal migration. (a) E17 cortices electroporated with indicated plasmids at E14 were stained for GFP and TO-PRO3. Scale bar: 100 μm. (b) Quantification of the percentages of GFP^+ cells in different cortical layers at E17. Error bars indicate the SEM of 3 different brains containing >300 neurons. (g) Quantification of the percentages of GFP^+ neurons with uni- or bipolar morphology, no process, and multiple (≥3) processes. Error bars indicate the SEM of 3 different brains containing >120 neurons. $^{**}P < 0.01$, $^{***}P < 0.001$ versus pSUPER, $^{\#\#}P < 0.05$, $^{\#\#\#}P < 0.001$ versus shCdk5; one-way ANOVA with post hoc Newman–Keuls test.

Supplementary Figure 9

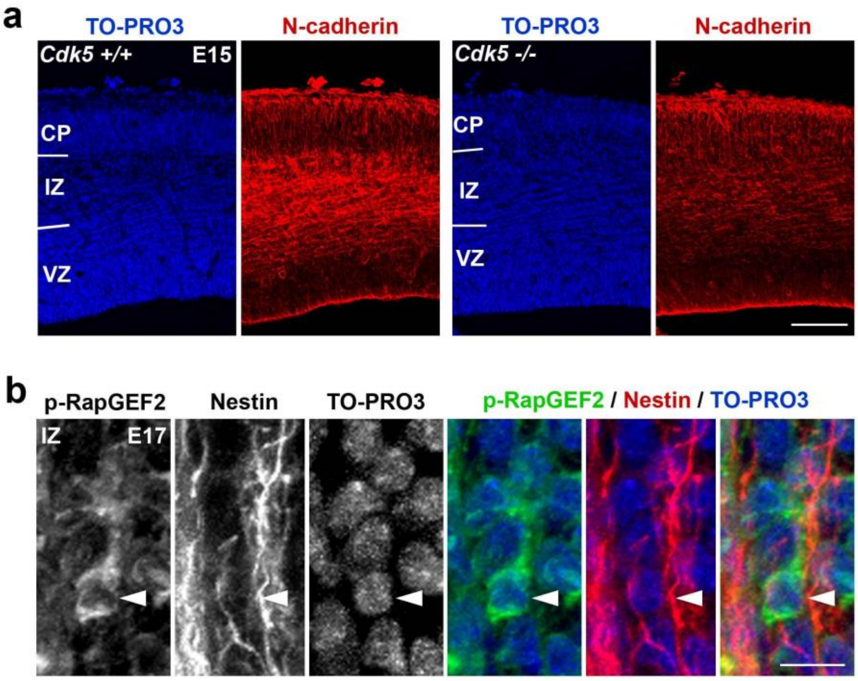

Supplementary Figure 9 Correlation between Cdk5-dependent RapGEF2 phosphorylation and N-cadherin–mediated adhesion. (a) Immunostaining for N-cadherin in the cortical wall of wild-type and Cdk5$^{-/-}$ embryos at E15. Coronal sections from E15 wild-type or Cdk5$^{-/-}$ brains were stained for N-cadherin and nuclear marker TO-PRO3. Scale bar: 10 μm. (b) An *in vivo* illustration of bipolar migrating neuron with strong phospho-RapGEF2 signals in the upper IZ that was associated Nestin-labeled radial glial fibers. E17 cortical sections were stained for p-RapGEF2, Nestin and TO-PRO3. Scale bar: 20 μm.

Supplementary Figure 10

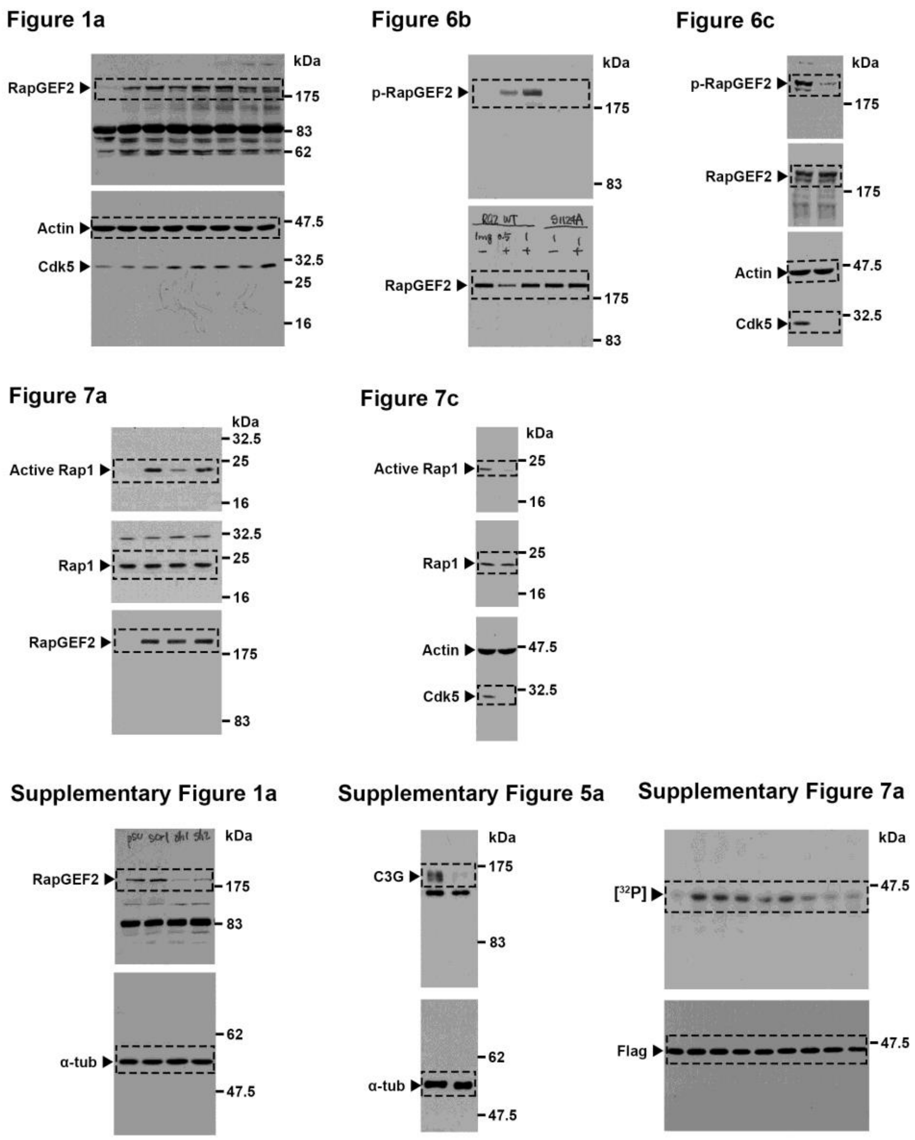

Supplementary Figure 10 Full length blots corresponding to figures 1a, 6b–c, 7a, 7c and supplementary figures 1a, 5a, 7a.
